# Visible-light driven regioselective synthesis, characterization and binding studies of 2-aroyl-3-methyl-6,7-dihydro-5*H*-thiazolo[3,2-*a*]pyrimidines with DNA and BSA using biophysical and computational techniques

**DOI:** 10.1038/s41598-021-01037-4

**Published:** 2021-11-11

**Authors:** Ranjana Aggarwal, Naman Jain, Shilpa Sharma, Prince Kumar, Gyan Prakash Dubey, Heerak Chugh, Ramesh Chandra

**Affiliations:** 1 Department of Chemistry, Kurukshetra University, Kurukshetra, Haryana 136119 India; 2 Council of Scientific and Industrial Research, National Institute of Science Communication and Policy Research, New Delhi, 110012 India; 3 Department of Chemistry, University of Delhi, New Delhi, 110007 India

**Keywords:** Drug discovery, Chemistry

## Abstract

In recent times, fused azaheterocycles emerged as impressive therapeutic agents. Binding studies of such azaheterocycles with biomolecules is an important subject for pharmaceutical and biochemical studies aiming at the design and development of new drugs. Fused heterocyclic scaffolds, such as thiazolopyrmidines have long been used in the pharmaceutical industry for the treatment of various diseases. In this study, we have accomplished a regioselective synthesis of 2-aroyl-3-methyl-6,7-dihydro-5*H*-thiazolo[3,2-*a*]pyrimidines by the reaction of tetrahydropyrimidine-2(*H*)-thione with α-bromo-1,3-diketones, generated in situ from 1,3-diketones and NBS, using visible light as an inexpensive, green and renewable energy source under mild reaction conditions with wide-ranging substrate scope. The regioisomer was characterized unambiguously by 2D-NMR [^1^H-^13^C] HMBC and [^1^H-^13^C] HMQC spectroscopy. In silico toxicity data analysis showed the low toxicity risks of the synthesized compounds. Computational molecular docking studies were carried out to examine the interaction of thiazolo[3,2-*a*]pyrimidines with calf-thymus DNA (ct-DNA) and Bovine Serum Albumin (BSA). Moreover, different spectroscopic approaches viz*.* steady-state fluorescence, competitive displacement assay, UV–visible and circular dichroism (CD) along with viscosity measurements were employed to investigate the binding mechanisms of thiazolo[3,2-*a*]pyrimidines with DNA and BSA. The results thus obtained revealed that thiazolo[3,2-*a*]pyrimidines offer groove bindings with DNA and showed moderate bindings with BSA.

## Introduction

It is well recognized that the thiazolo[3,2-*a*]pyrimidine; a fused heterocyclic system consisting of six-membered pyrimidine and five-membered thiazole rings with bridgehead nitrogen atom is a privileged motif found in numerous natural products and various synthetic compounds. In the recent times, it has emerged as a potential synthetic area in heterocyclic synthesis due to its widespread applications among as acetylcholinesterase inhibitor^1^, calcium antagonist^2^, cytotoxic agent^3^, antitubercular, CDC25 phosphatase inhibitor^4^, antidepressant^5^ as well as in polymerization reactions as co-polymer^6–8^. Although short of literature, the synthesis of thiazolo[3,2-*a*]pyrimidine nucleus has been reported by the sodium acetate catalyzed reaction of tetrahydropyrimidine-5(*H*)-2-thione with α-halo carbonyl compounds^1,5,9^ in acetic acid reflux, 1,1,1,3,4,4,5,5,5-nonafluoro-2-(trifluoromethyl)pent-2-ene^10^ in triethylamine catalyzed acetonitrile reflux and by the reaction of aromatic aldehydes with 2-aminothiazole and malononitrile/diethyl malonate/ethyl 2-cyanoacetate in piperidine catalyzed ethanolic reflux^11,12^. These methods use organic bases as catalysts and in most of the reactions, the product obtained is thiazolo[3,2-*a*]pyrimidin-3-ol which needs to be dehydrated to give the required thiazolo[3,2-*a*]pyrimidine nucleus.

Heterocyclic ring-containing compounds are endowed with interesting biomolecular recognition properties^13^. In recent times, ex-vivo studies of fused azaheterocycles with biomolecules have emerged as a powerful tool to objectify design and development of novel chemotherapeutic agents. Some chemotherapeutic agents impart their pharmacological effect by binding with DNA or cleave DNA helix^14,15^, in turn inhibiting the division of cancer cells. Thus it’s of vital importance to study the binding mechanism of these small molecules with DNA using various biophysical and computational approaches^16^.

The two most important proteins that have been used for protein-drug interaction studies are Bovine Serum Albumin (BSA) and Human Serum Albumin (HSA). Both the proteins play a vital role in drug transportation in the blood to a particular target affecting its ADMET profile however, the former is a bovine plasma protein and makes an excellent replacement for HSA because of its shared homology^17^.

**Figure 1:**
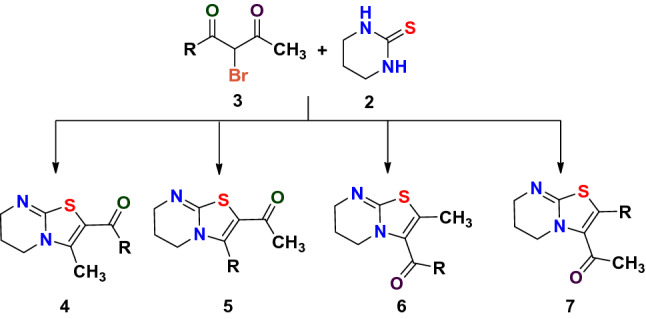
Showing four possible products on the reaction of α-bromo-1,3-diketone with terahydropyrimidine-2(*H*)-thione.

Considering the immense biological importance of thiazolo[3,2-*a*]pyrimidines and in continuation to our previous studies related to the regioselectivity study of the reaction between 1,3-diketones and various binucleophiles^18–20^, it was envisaged to undertake the study of regioselectivity pattern in the reaction between 1,3-diketones **1** and tetrahydropyrimidine-2(*H*)-thione **2** to synthesize the thiazolo[3,2-*a*]pyrimidine nucleus. For this purpose, the unsymmetrical 1,3-diketone **1** was reacted with NBS to produce α-bromo-1,3-diketone **3** which was used in situ for condensation with tetrahydropyrimidine-2(*H*)-thione **2** in presence of visible light. As evident that the α-bromo-1,3-diketone **3** has three electrophilic sites, so after the expulsion of bromine, possibility of formation of four products exists; 2-aroyl-3-methyl-6,7-dihydro-5*H*-thiazolo[3,2-*a*]pyrimidines **4**, (2-methyl-6,7-dihydro-5*H*-thiazolo[3,2-*a*]pyrimidin-3-yl)aryl methanone **5**, 1-(2-aryl-6,7-dihydro-5*H*-thiazolo[3,2-*a*]pyrimidin-3-yl)ethan-1-one **6** and 1-(3-aryl-6,7-dihydro-5*H*-thiazolo[3,2-*a*]pyrimidin-2-yl)ethan-1-one **7** depending on the electrophilicity difference between the two carbonyl carbons as shown in Fig. 1.

However, to our astonishment, the reaction yielded only a single regioisomer as 2-aroyl-3-methyl-6,7-dihydro-5*H*-thiazolo[3,2-*a*]pyrimidines **4**. The structure of the regioisomer was unambiguously characterized by heteronuclear 2D NMR [(^1^H-^13^C) HMBC, (^1^H-^13^C) HMQC] spectroscopy. Interaction studies of thiazolo[3,2-*a*]pyrimidines with DNA and BSA using computational approach and various spectroscopic techniques such as UV–Vis absorption spectroscopy, steady-state fluorescence, circular dichroism were carried out to explore the interaction mechanism. Other practices such as competitive displacement assay and viscosity measurements were also employed to understand whether the ligand molecule binds with ct-DNA in groove region or intercalates into the base pairs of double-helix DNA.

## Results and discussion

### Chemistry

The α-bromo-1-phenylbutane-1,3-dione was prepared by the reaction of NBS with phenylbutane-1,3-dione at room temperature and was used in situ. The reaction optimization started with the reaction of tetrahydropyrimidine-2(*H*)-thione **2** with α-bromo-1-phenylbutane-1,3-dione **3a** in various solvents like DCM, ethanol, methanol at room temperature. It was observed that although, the reaction goes to completion in about 1–2 h, the reaction yields were very poor (45%, 43% and 55% respectively). Being an inexpensive, clean, abundant and renewable source of energy for green chemical reactions^21^, visible light has emerged as a boon for organic transformations in past few years. Besides, it has become a powerful tool to construct new chemical bonds and hereby has gained wide applications in organic synthesis. Taking into consideration the emerging use of widely available visible light, the reaction between α-bromo-1-phenylbutane-1,3-dione **3a** and tetrahydropyrimidine-2-thione **2** was investigated using different solvents under visible light reaction conditions. α-Bromo-1-phenylbutane-1,3-dione **3a** used was prepared by the solvent free monobromination reaction of phenylbutane-1,3-dione with NBS in dry pestle and mortar^22^. It was observed that the reaction proceeded smoothly in DCM, ethanol and methanol but the best results are obtained when using ethanol and water mixture in 4:1 ratio by volume. The EtOH:H_2_O system may be ideal to increase the solubility of reactants and hence to increase the reaction yield. Also, the higher static permeability of water contributes to stabilization of intermittent transition states in the reaction. Further, the hydrophobic nature of reactants in water leads to increase in number of collisions which along with visible light source contribute to increase in ground state energy and reaction rates. Water as co-solvent improves the efficiency of reaction with easy product isolation^23^.

The reaction goes to completion with single product formation within 15–20 min as indicated by TLC with 85% reaction yields. The elementary studies revealed the presence of a single peak in infrared region at 1587 cm^−1^ due to stretching of the C=O group indicating the complete consumption of 1,3-diketone. Further, ^1^H-NMR studies showed a single peak at δ 2.25 ppm integrating for three protons of the methyl group, two triplets at δ 3.77–3.75 ppm and δ 3.53–3.51 ppm for protons at C-5 and C-7, a quintet for two protons at C-6 along with five protons of phenyl ring confirmed the successful condensation of two reactants to form 3-methyl-6,7-dihydro-5*H*-thiazolo[3,2-*a*]pyrimidin-2-yl)aryl methanones. Apart from this, the HRMS and elemental analysis were also in accordance with the structure proposed.

**Figure 2:**
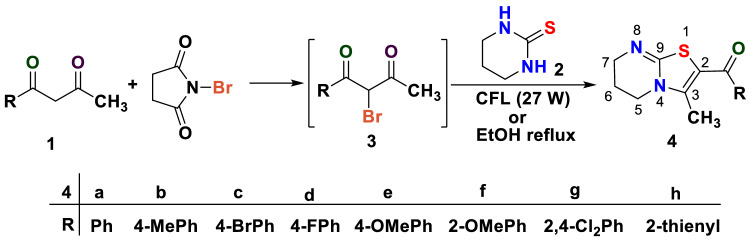
One-pot regioselective synthesis of 2-aroyl/heteroaroyl-3-methyl-6,7-dihydro-5*H*-thiazolo[3,2-*a*]pyrimidine derivatives.

After the reaction conditions were optimized, differently substituted aryl and heteroaryl 1,3-diketones **1a–h** were employed to react with tetrahydropyrimidine-2-thione **2** to generate a series of 2-aroyl/heteroaroyl-3-methyl-6,7-dihydro-5*H*-thiazolo[3,2-*a*]pyrimidines **4a–h** where all the employed diketones **1a–h** displayed excellent selectivity and the products **4a–h** were obtained in good yields (Fig. 2). The summary of products obtained with the reaction yields is summarized in Table 1.

**Table 1:** Showing the product formation along with the reaction yields.

S. no.	Diketone	Product	Yield (%)
1			85
2			82
3			88
4			83
5			82
6			81
7			84
8			78

The elementary data along with one-dimensional NMR values was not sufficient to mark the product among the four regioisomers 2-aroyl/heteroaroyl-3-methyl-6,7-dihydro-5*H*-thiazolo[3,2-*a*]pyrimidines **4**, (2-methyl-6,7-dihydro-5*H*-thiazolo[3,2-*a*]pyrimidin-3-yl)(aryl/heteroaryl)methanone **5**, 1-(2-aryl/heteroaryl-6,7-dihydro-5*H*-thiazolo[3,2-*a*]pyrimidin-3-yl)ethan-1-one **6** and 1-(3-aryl/heteroaryl-6,7-dihydro-5*H*-thiazolo[3,2-*a*]pyrimidin-2-yl)ethan-1-one **7** as the values are very close and disconcerting. So, in order to end the ambiguity and to establish the structure, 2D-NMR [(^1^H-^13^C) HSQC and (^1^H-^13^C) HMBC)] of compound **4a, 4f** and **4g** was carried out and the results are summarized in (Supplementary data; Page: S13–18).

**Figure 3:**
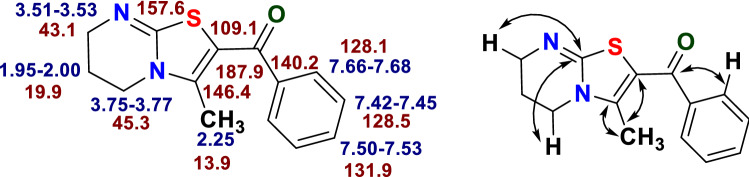
Showing ^1^H-NMR (in blue) and ^13^C-NMR (in brown) chemical shifts of compound **4a** along with the correlation illustration.

The (^1^H-^13^C) HMBC and (^1^H-^13^C) HMQC of the compound 2-benzoyl-3-methyl-6,7-dihydro-5*H*-thiazolo[3,2-*a*]pyrimidine **4a** revealed the cross-peaks of methyl protons (δ 2.25 ppm) with C-2 (δ 109.1 ppm) and C-3 (δ 146.4 ppm). Similarly, the cross-peaks between carbonyl carbon at δ 187.9 ppm and 2′/6′-H protons (δ 7.68–7.66 ppm) of the aryl group confirmed the presence of carbonyl group with phenyl ring abolishing the presence of acetyl group and hence the regioisomers **5** and **7**. Hence, the structure can out rightly be allocated as 2-benzoyl-3-methyl-6,7-dihydro-5*H*-thiazolo[3,2-*a*]pyrimidine **4a**. The 2D NMR correlation results attained for compound **4a** along with the ^1^H and ^13^C-NMR values are illustrated in Fig. 3.

**Figure 4:**
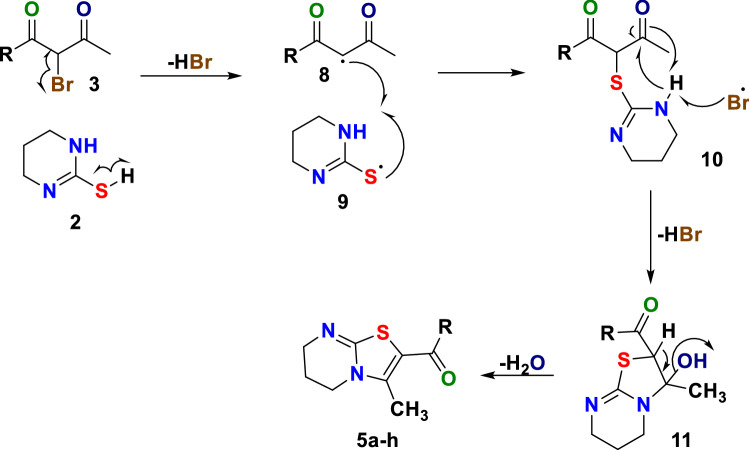
Showing a proposed mechanism for the synthesis of thiazolo[3,2-*a*]pyrimidines **4a–h**.

The possible mechanistic path for the regioselective synthesis of 2-aroyl/heteroaroyl-3-methyl-6,7-dihydro-5*H*-thiazolo[3,2-*a*]pyrimidine **4a–h** is depicted in Fig. 4. Visible light mediated reaction initiates by the homonuclear fission of C–Br bond of α-bromodiketone **3** and S–H bond of tetrahydropyrimidine-2-thione **2** to generate free radicals **8** and **9** which combine to form S-alkylated open chain structure **10**. The bromine and hydrogen free-radicals generated in the step facilitate the homonuclear fission of N–H and C=O bond respectively of intermediate **10** to generate two new free radicals; amine and carbonyl group adjacent to methyl group; the intramolecular combination of these free radicals generated the (3-hydroxy-3-methyl-2,3,6,7-tetrahydro-5*H*-thiazolo[3,2-*a*]pyrimidin-2-yl)(aryl/heteroaryl)methanone **11** which undergoes dehydration to yield 2-aroyl/heteroaroyl-3-methyl-6,7-dihydro-5*H*-thiazolo[3,2-*a*]pyrimidines **4** as product compatibility to our previous results^18,19^. The regioselective route followed in the reaction can be explained based on less steric hindrance and crowding on acetyl group as compared to aroyl/heteroaroyl group making the former more susceptible to react.

### In-silico studies

#### Lipinski’s rule of five

The Osiris program was used to calculate Lipinski’s rule of five that helps in the prediction of the oral bioavailability of the drug molecules. cLogP value of a compound i.e. algorithm of partition coefficient between n-octanol and water is a well-established measure of the compound’s hydrophilicity. cLogP values must not be higher than 5.0 as it cause the poor absorption and permeation. The compounds having total polar surface area (TPSA) > 140 are passively absorbed and thus have low oral bioavailability^24^. Compounds **4a–h** have TPSA value ranging from 57.97 to 86.21 and low value of partition coefficient (2.21–3.49) as well (Table 2). Outcomes revealed that all the compounds go along with rules of oral bioavailability without any violation, indicating that these compounds have potential to be possible drug molecules.

**Table 2:** Calculated Lipinski’s rule of five for tested compounds **4a–h**.

C. no.	TPSA	MW	cLogP	nHBA	nHBD	RB
4a	57.97	258	2.28	3	0	2
4b	57.97	272	2.62	3	0	2
4c	57.97	337	3.00	3	0	2
4d	57.97	276	2.38	3	0	2
4e	67.20	288	2.21	4	0	3
4f	67.20	288	2.21	4	0	3
4g	57.97	327	3.49	3	0	2
4h	86.21	264	2.15	3	0	2

*TPSA* Total polar surface area, *MW* molecular weight, *cLogP* calculated lipophilicity, *nHBA* number of hydrogen bond acceptors, *nHBD* number of hydrogen bond donors, *RB* rotatable bonds.

### Calculation of toxicities, drug-likeness, and drug score profiles

We have also analysed the overall toxicity of the synthesized derivatives by using Osiris program. The predicted values of toxicity, drug-likeness and drug score for compounds **4a–h** are shown in Table 3. Drug score is a useful parameter which combines to cLog P, Log S, molecular weight, drug-likeness and toxicity risks to give one value. In general, drug score of ≥ 0.4 for a compound makes it a potential drug candidate with good safety and efficiency^25^. In silico studies showed that all the target compounds have log S values above − 3 and thus can have good aqueous solubility. Green colour indicated that target compounds **4a–h** showed no in silico toxicity risks (Table 3). It is evident from the results that the compounds **4g** exhibited high drug-likeness value than other derivatives.

**Table 3:** Toxicity risks, drug-likeness, and drug score for tested compounds 4**a–h**.

C. no.	Log S	Drug-likeness	Drug score	Toxicity risk			
				Mutagenicity	Tumorigenicity	Irritating effects	Reproductive effects
**4a**	− 3.80	4.82	0.94				
**4b**	− 4.15	4.29	0.92				
**4c**	− 4.64	2.14	0.84				
**4d**	− 4.12	5.35	0.93				
**4e**	− 3.82	5.30	0.93				
**4f**	− 3.82	5.06	0.93				
**4g**	− **5.28**	**5.99**	0.86				
**4h**	− 3.81	5.41	0.94				

*LogS*-Solubility parameter,  Low toxicity risk.

### Molecular reactivity analyses

**Figure 5:**
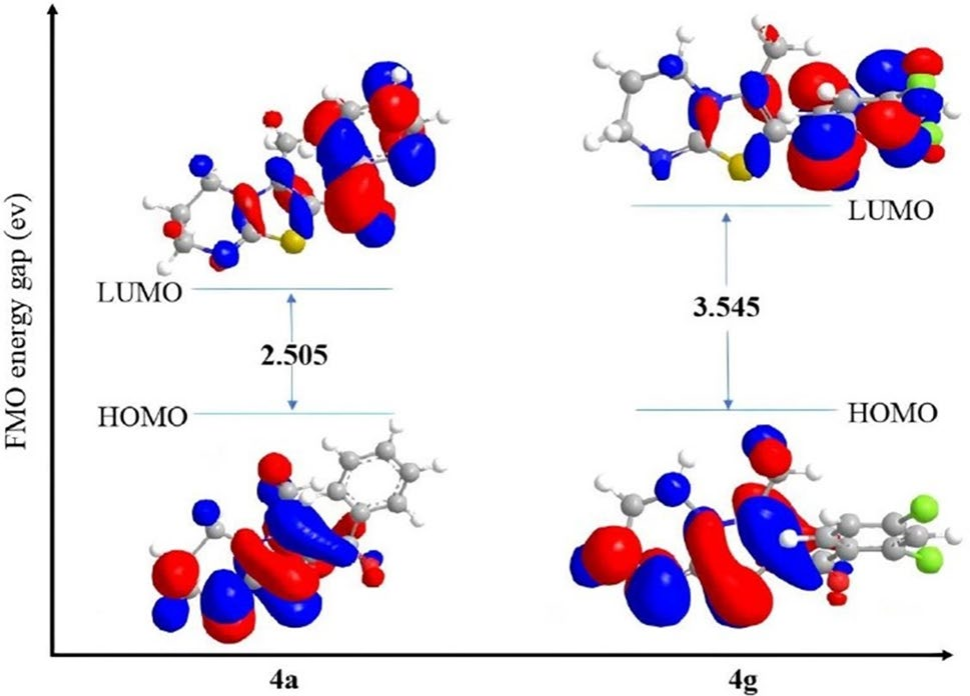
Frontier orbital and energy gap of frontier orbitals of compound **4a** and **4g.** The figure was generated using PerkinElmer Chem3D 15.0.0.106. (https://perkinelmer-chemdraw-professional.software.informer.com/15.0/).

It is a well-known fact that reaction among molecules takes place due to transfer of electrons between highest occupied molecular orbital (HOMO) and lowest unoccupied molecular orbital (LUMO) also known as frontier orbitals. Study of these frontier orbitals helps to understand the molecular reactivity. If a molecule accepts an electron it always occupy LUMO, lower the energy of LUMO easier it will be for a molecule to accept the electron^26^. Likewise, an electron in HOMO with higher energy will always be preferred for donation. We implement a theoretical study by using mm + force field energy (PerkinElmer Chem3D 15.0.0.106) minimization for calculating the molecular orbital energies (HOMO and LUMO). Table 4 depicted the energies of frontier orbitals of aryl derivatives of thiazolo[3,2-*a*]pyrimidine. Lower energy gap (∆E) between HOMO–LUMO indicates higher reactivity of compound, while higher energy gap (∆E) between orbitals indicates lower reactivity of the compound^27^. Data obtained showed that compound **4a** is the most reactive whereas, compound **4g** is the least among thiazolo[3,2-*a*]pyrimidines. The pictorial presentation of frontier orbitals along with the energy gap (∆E) represented in the Fig. 5.

**Table 4:** Frontier orbitals energy and global reactivity descriptors of compound 4a–g.

Comd	E_HOMO_ (ev)	E_LUMO_ (ev)	∆E	IP	EA	Ƞ	µ	S	χ	ω
4a	− 6.314	− 3.809	2.505	6.314	3.809	1.252	− 5.061	0.399	5.061	10.229
4b	− 6.304	− 3.589	2.715	6.304	3.589	1.357	− 4.946	0.368	4.946	9.013
4c	− 6.284	− 3.257	3.027	6.284	3.257	1.513	− 4.770	0.330	4.770	7.519
4d	− 6.281	− 3.059	3.222	6.281	3.059	1.611	− 4.670	0.310	4.670	6.768
4e	− 6.276	− 3.440	2.836	6.276	3.440	1.418	− 4.858	0.352	4.858	8.321
4f	− 6.288	− 3.225	3.063	6.288	3.225	1.531	− 4.756	0.326	4.756	7.387
4g	− 6.261	− 2.716	3.545	6.261	2.716	1.772	− 4.488	0.282	4.488	5.683

Ionization potential (IP) and electron affinity (EA) values are associated with frontier orbitals and were calculated according to Koopman theorem using following relation:

$$\Delta {\text{E }} = {\text{ E}}_{{{\text{LUMO}}}} - {\text{E}}_{{{\text{HOMO}}}} ,$$

$${\text{IP}} = - {\text{E}}_{{{\text{HOMO}} }} \, {\text{ and }}\,{\text{EA}} = - {\text{E}}_{{{\text{LUMO}}}} .$$

Furthermore, Global reactivity descriptors chemical hardness (η) and softness (S), electronegativity (χ), electrophilic index (ω) and chemical potential (µ) as described by Pearson and Parr^28^ were calculated using following equations and are depicted in the Table 4

$$\mathrm{Chemical hardness is designated as }\left(\eta \right)= \frac{\left(IP-EA\right)}{2},$$

$$\mathrm{Chemical softness is designated as }\left(\mathrm{S}\right)= \frac{1}{2\eta },$$

$$\mathrm{Electronegativity is designated as }\left(\upchi \right)= \frac{\left(IP+EA\right)}{2},$$

$$\mathrm{Chemical potential is designated as }\left(\upmu \right)= \frac{-\left(IP+EA\right)}{2},$$

$$\mathrm{Electrophilic index is designated as }\left(\upomega \right)= \frac{{\mu }^{2}}{2\eta }.$$

It is evident that compound **4g** with highest energy gap (∆E), has the low polarizability and most kinetically stable among these aryl derivatives. With lowest ionization potential and electrophilicity index compound **4g** can easily donate electrons to bind with biomolecules through non-covalent interactions. Other physicochemical parameters such as chemical potential, hardness, softness and electronegativity also support the binding behaviour of the compound with biomolecules^29^.

### Molecular docking studies

#### For DNA

**Figure 6:**
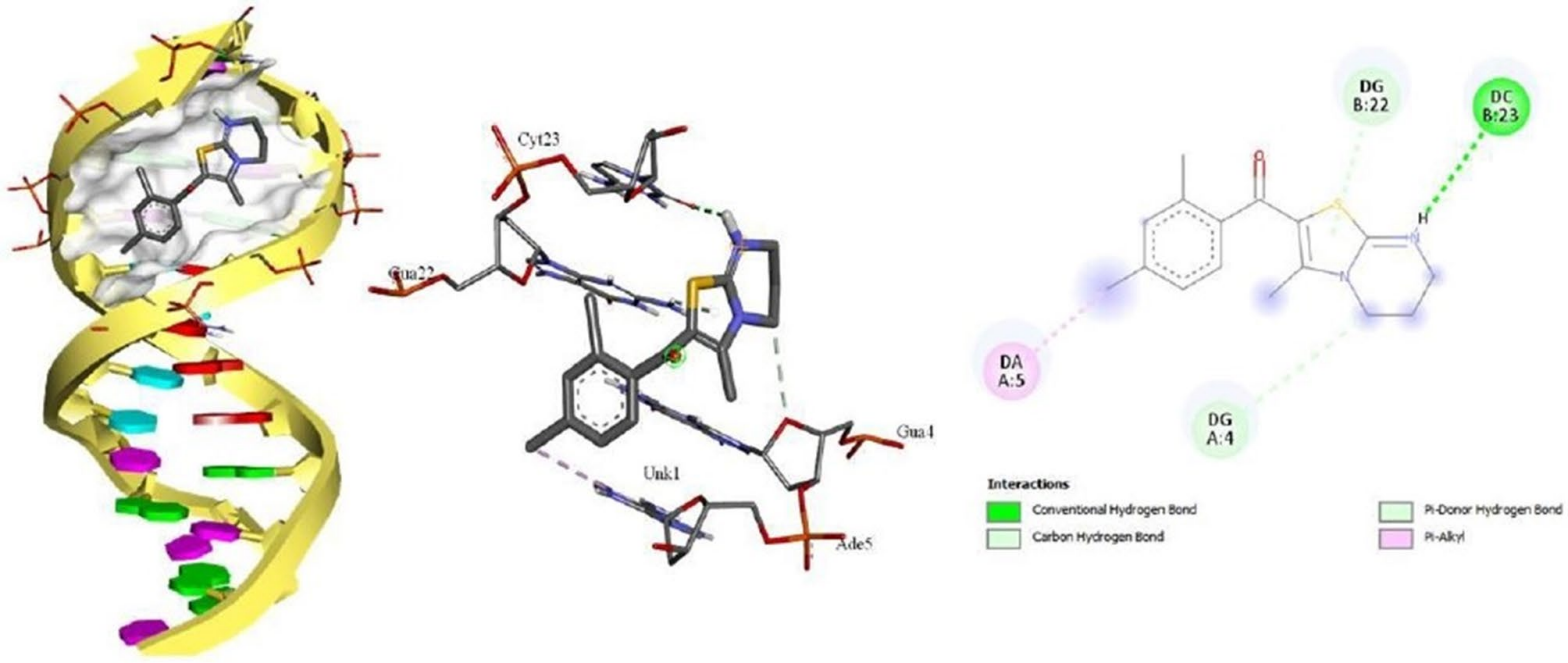
Docking pose of compound **4g** and interactions with ct-DNA. The figure was generated using BIOVIA Discovery Studio Visualizer (DSV) (https://discover.3ds.com/discovery-studio-visualizer-download).

The Molecular docking tool is an impressive computational practice that estimates the interactions of the molecules in the 3D spatial arrangement with a purposive receptor. Docking results offer in-depth information on the interactions of biomolecules (protein, nucleic acids, etc.) with small organic molecules at the atomic level by studying the root mean square deviances and protein/nucleic acid confirmations. To find the best binding derivative among the novel synthesized thiazolopyrimidine derivatives, in silico molecular docking studies were carried out. Outcomes of this study showed that among the synthesized thiazolopyrimidine derivatives, compound with 2,4-dichlorosubstitution **4g** binds with DNA dodecamer d(CGCGAATTCGCG)_2_ (PDB ID:1BNA) more efficiently than other derivatives with a maximum docking score of − 7.5 kcal/mol (Table 5). Interaction positions were examined by docking analysis in BIOVIA Discovery Studio Visualizer (DSV) which shows that 2,4-dichloro derivative binds to ct-DNA at the Guanine-Cytosine rich region in minor grove through non-covalent interactions such as; Van der Waal’s interactions, conventional hydrogen bindings, and various hydrophobic interactions (Fig. 6).

**Table 5:** Binding affinities of thiazolo[3,2-*a*]pyrimidine derivatives **4a–h** for DNA/BSA.

Compound no	R	Affinity for BSA (kcal/mol)	Affinity for DNA (kcal/mol)
4a	Ph	− 7.8	− 6.6
4b	4-MePh	− 7.8	− 6.9
4c	4-BrPh	− 7.7	− 6.8
4d	4-FPh	− 7.8	− 6.7
4e	4-OMePh	− 7.3	− 6.9
4f	2-OMePh	− 7.1	− 6.6
**4g**	**2,4-diClPh**	**− 8.0**	**− 7.5**
4h	2-Thienyl	− 6.7	− 6.0

#### For BSA

**Figure 7:**
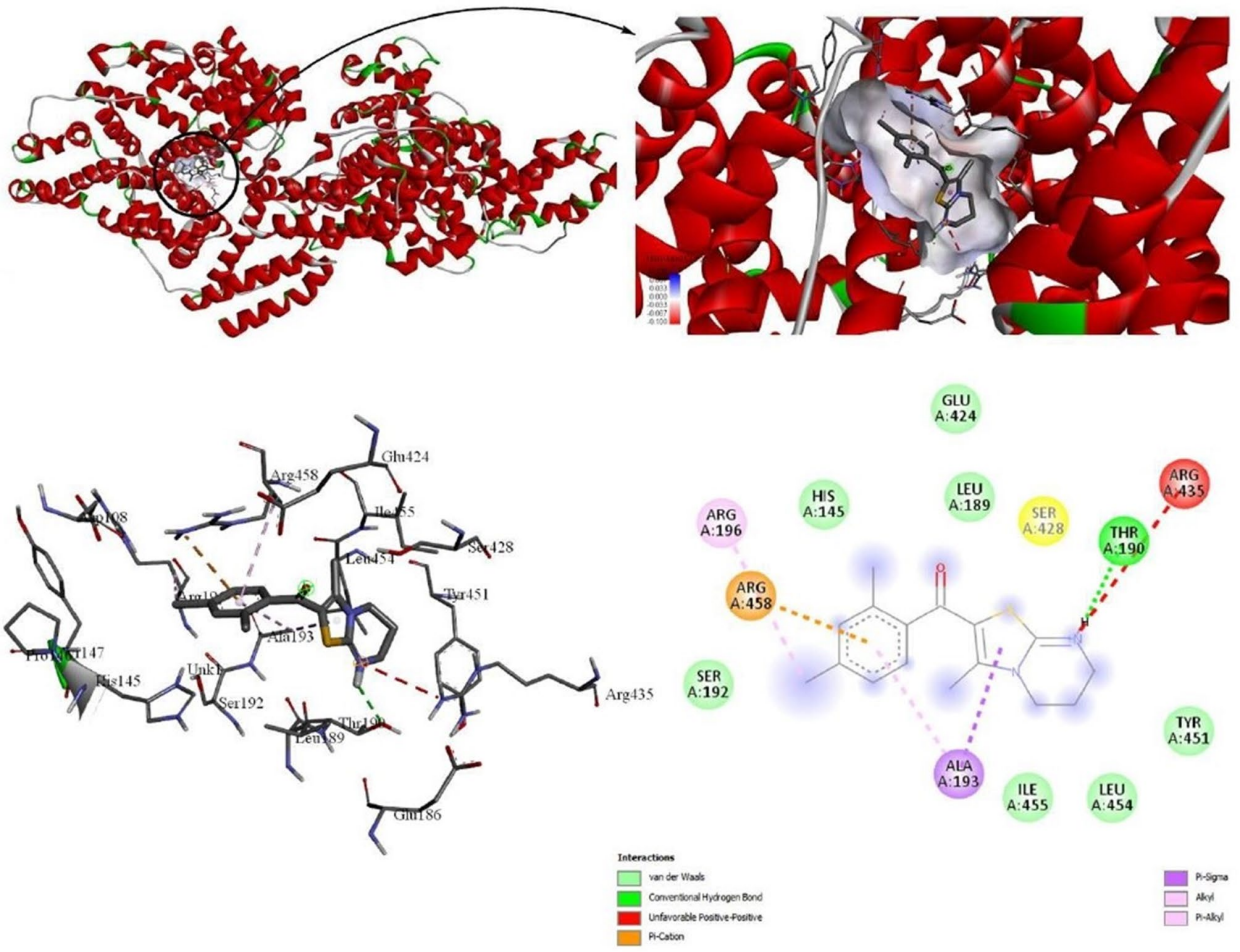
A 2D/3D plots of **4g** interacted in the subdomain of chain A of BSA. The figure was generated using BIOVIA Discovery Studio Visualizer (DSV) (https://discover.3ds.com/discovery-studio-visualizer-download).

In this examination, all the synthesized derivatives were processed with albumin protein (BSA) in AutoDock vina and result revealed that again compound **4g** with 2,4-dichloro substitution binds effectively with BSA with the highest binding affinity − 8.0 kcal/mol (Table 5). Further DSV analyzer showed that **4g** interacts with serum albumin in the active pocket of chain A. The docked complex interacting with amino acids (Arg:196, Arg:435, Arg:458, Thr:190 and Ala 193) is shown in the 2D and 3D plots (Fig. 7). 2D representation showed that the **4g** interacts with various amino acids through Van der Waal’s forces, hydrogen bonding and various hydrophobic interactions.

### Binding studies of thiazolo[3,2-*a*]pyrimidines with DNA and BSA

#### UV–visible absorption spectroscopy studies

In recent times, UV–visible spectroscopy emerged as an important and valuable tool for biomolecule-drug interaction studies due to its simple operation and versatility^30^. The interactions of DNA/BSA with ligands are characterized by variation in the intensity or wavelength shift in UV–visible spectra resulted from perturbation of the biomolecule’s electronic environment^31^. This transformation in the UV–visible absorption spectra of DNA/BSA at maximum wavelength is very vital since this change tells whether a ligand interacts with biomolecule or not, also in the case of DNA it designates the mode by which DNA interacts with drug (groove or intercalators)^32^. Based on the studies, red shift or intensity increment is observed in the UV–visible spectra when the drug interacts with DNA via grove-binding mode whereas blue shift or intensity decrement is observed when a drug intercalates in the base pairs of DNA^33^.

**Figure 8:**
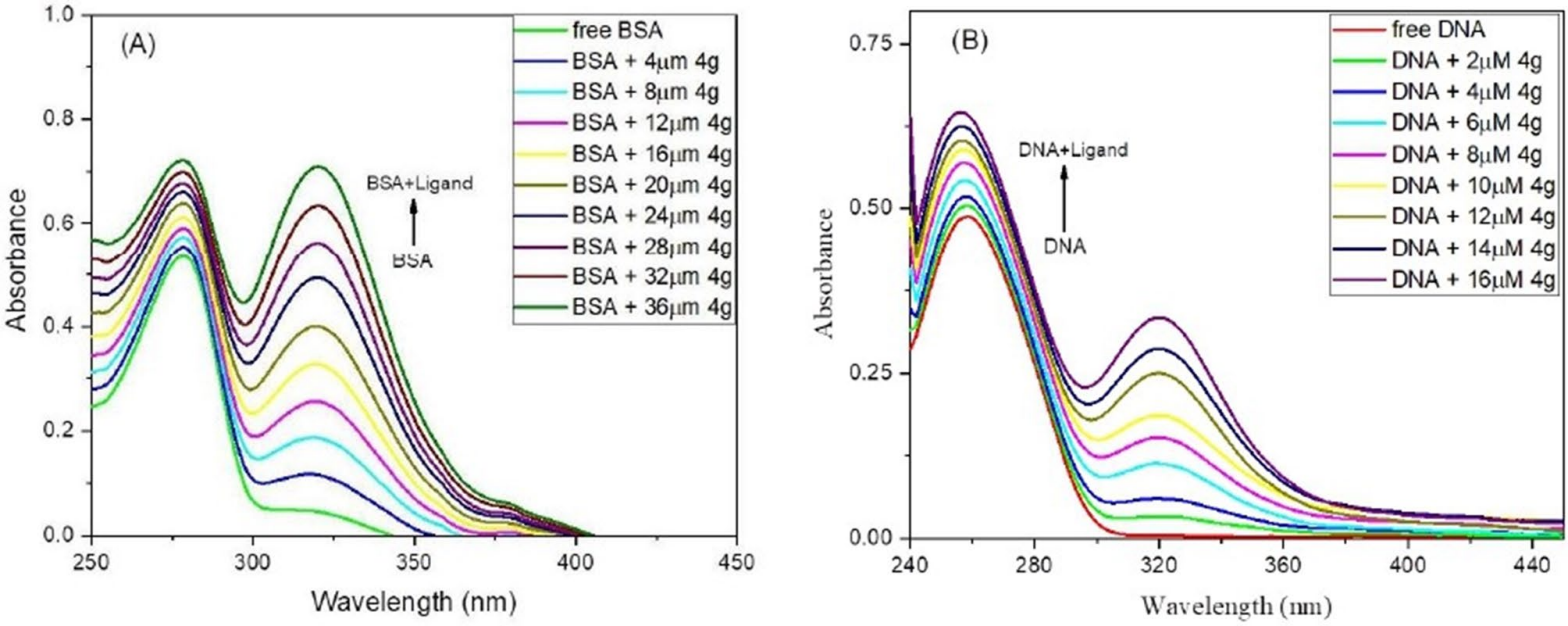
(**A)** UV–visible spectra of BSA-ligand complex system at increasing concentrations of **4g** (0–36 µM) at constant BSA concentration of 15 µM (**B**) Absorption spectra of ct-DNA-**4g** complex at a varying concentration of **4g** (0–16 µM) by keeping the concentration of DNA constant at 72 µM in physiological pH 7.2 of Tris–HCl buffer at room temperature.

Two absorption experiments were performed, in the first experiment UV–visible spectra of biomolecules (DNA and BSA) were investigated in the wavelength range of 220–450 nm for BSA and 230–440 nm for DNA in the absence and presence of **4g** at a varying concentration ranging from 0 to 36 µM and (0–16 µM) for BSA and DNA, respectively at room temperature. Initially, absorption spectra of DNA/BSA in the absence of **4g** displayed their characteristic peaks at 278 nm in case of BSA accredited to π–π* transition due to the presence of aromatic amino acids viz*.* tryptophan, tyrosine and phenylalanine^34^ on the protein chain surface and at 260 nm in case of DNA. Finally, spectra of DNA/BSA-**4g** complex were inspected with an increasing concentration of **4g.** The intensity of the BSA spectra goes on increasing after each titration due to the transformation in the environment around aromatic amino acids Trp, Phe and Tyr (Fig. 8A), also an increase in the absorption spectra value (hyperchromic shift) of DNA with an increasing amount of **4g** was observed indicating preferably the grove binding of **4g** with DNA^35^ (Fig. 8B). This increase in the absorption maxima of DNA/BSA on increasing the amount of **4g** designates an interaction between DNA/BSA and thaizolopyrimidine ligand.

**Figure 9:**
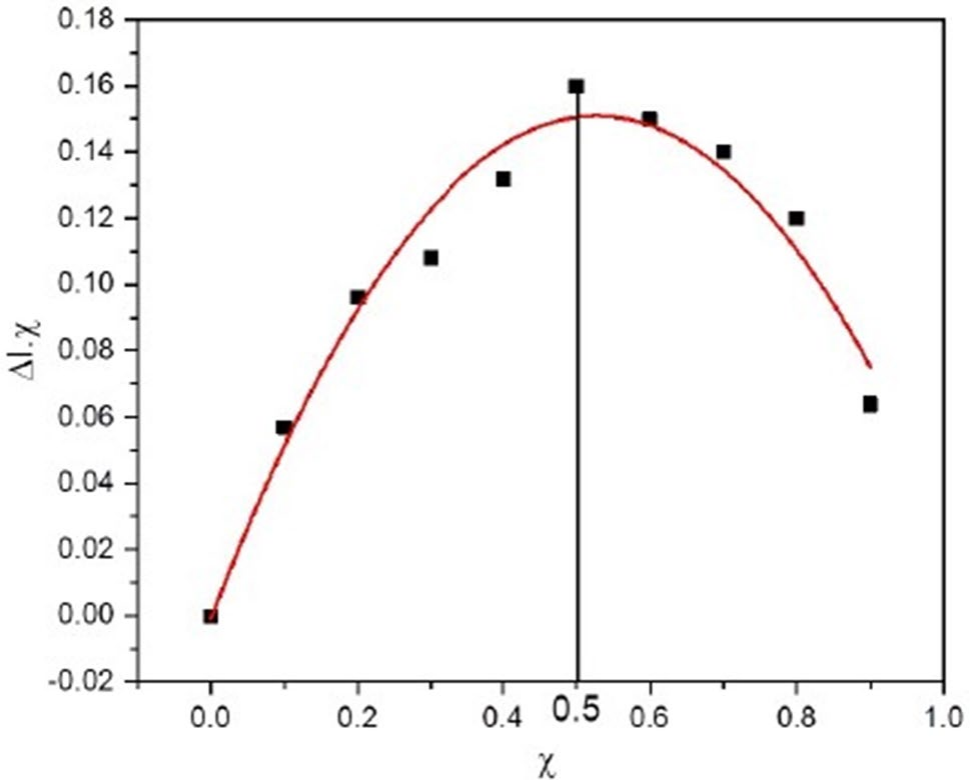
Job’s plot display binding stoichiometry of BSA with **4g.**

In second experiment, ten different solutions were prepared with different volumes of BSA and **4g** fixed at a constant concentration 20 µM. Using this process, the ratio by which BSA protein interacts with a ligand can be easily determined employing Job’s plot using UV–visible titrations. In Job’s plot, the mole fraction of ligand **4g** was plotted against corrected absorbance at 278 nm. The maxima thus computed using Job’s plot between absorbance at 278 nm and mole fraction of ligand **4g** helped in establishing the binding stoichiometry (Fig. 9). The maxima was observed at 0.5 on the axis indicating that BSA binds with ligand **4g** in 1:1 binding stoichiometry^36^.

#### Fluorescence quenching studies

**Figure 10:**
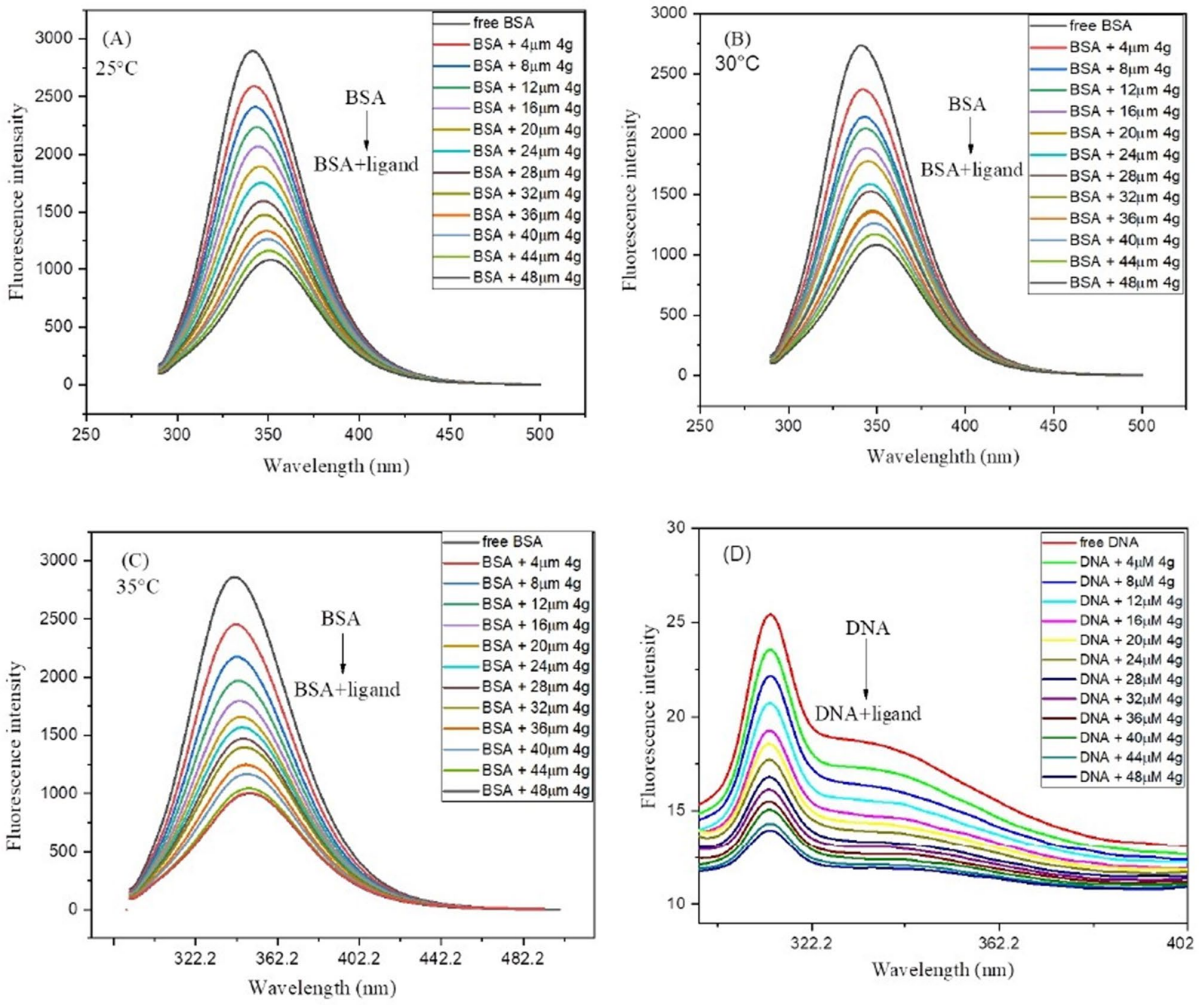
Fluorescence spectra of pure DNA/BSA and DNA/BSA-**4g** complex at a variable concentration of **4g** (0–48 µM) (**A**) BSA spectra at 25 °C; (**B**) BSA spectra at 30 °C; (**C**) BSA spectra at 35 °C; (**D**) DNA spectra at 25 °C.

To further investigate the mode of interaction between biomolecules (DNA/BSA) and ligand **4g**, the fluorescence studies were carried out by keeping the concentration of DNA and BSA constant. The spectra were recorded both in the absence and presence of the compound **4g** in the spectral range of 290–500 nm at the fix excitation wavelength of 280 nm in case of BSA and 300–500 nm by setting the excitation wavelength at 290 nm in case of DNA. During the DNA/BSA-ligand interactions, a change in DNA/BSA conformation was encountered due to alteration in the microenvironment of the macromolecule which can be examined easily with the help of steady-state fluorescence spectroscopy. A maximum at 342 nm was observed in the case of BSA whereas a maximum of nearly 314 nm was detected in the case of DNA. The DNA/BSA emission spectra were successively quenched by increasing the suitable amount of drug **4g** (0-48 µM) at a different range of temperatures (25 °C, 30 °C, 35 °C) in case of BSA (Fig. 10A–C) and 25 °C in case of DNA (Fig. 10D).

The reduction in the intensity of DNA/BSA spectra after the addition of drug **4g** was appeared due to the quenching mechanism which is mainly separated into three classes viz*.* static quenching, dynamic quenching and mixed quenching (combination of both static and dynamic quenching). In general, static quenching is a ground state phenomenon that occurs due to the formation of a complex between fluorophore (DNA/BSA) and quencher **4g** in the ground state, whereas; dynamic quenching is an exciting state phenomenon that arises due to collisions between fluorophore and quencher in the excited state^37^. How quenching proceeds can be easily identified by calculating the quenching constant *Kq* using the Stern–Volmer equation^38^ (Eq. 1).

1 $$\frac{Fo}{F}= 1 + Ksv\left[\mathrm{Q}\right]= 1 + Kq\mathrm{ \tau_o }\left[\mathrm{Q}\right].$$

The symbol F_o_ denotes the fluorescence intensity of pure DNA/BSA, F refers to the fluorescence intensity of DNA/BSA in the existence of **4g** at the various concentrations (0–48 µM). [Q] is a molar concentration of **4g**. τ_o_ denotes the average lifetime of DNA/BSA. *K*_*SV*_ and *Kq* are the Stern Volmer constant and quenching constant respectively. Quenching constant *Kq* was calculated by presuming average lifetime^39^ τ_o_ = 10^–8^ s.

**Figure 11:**
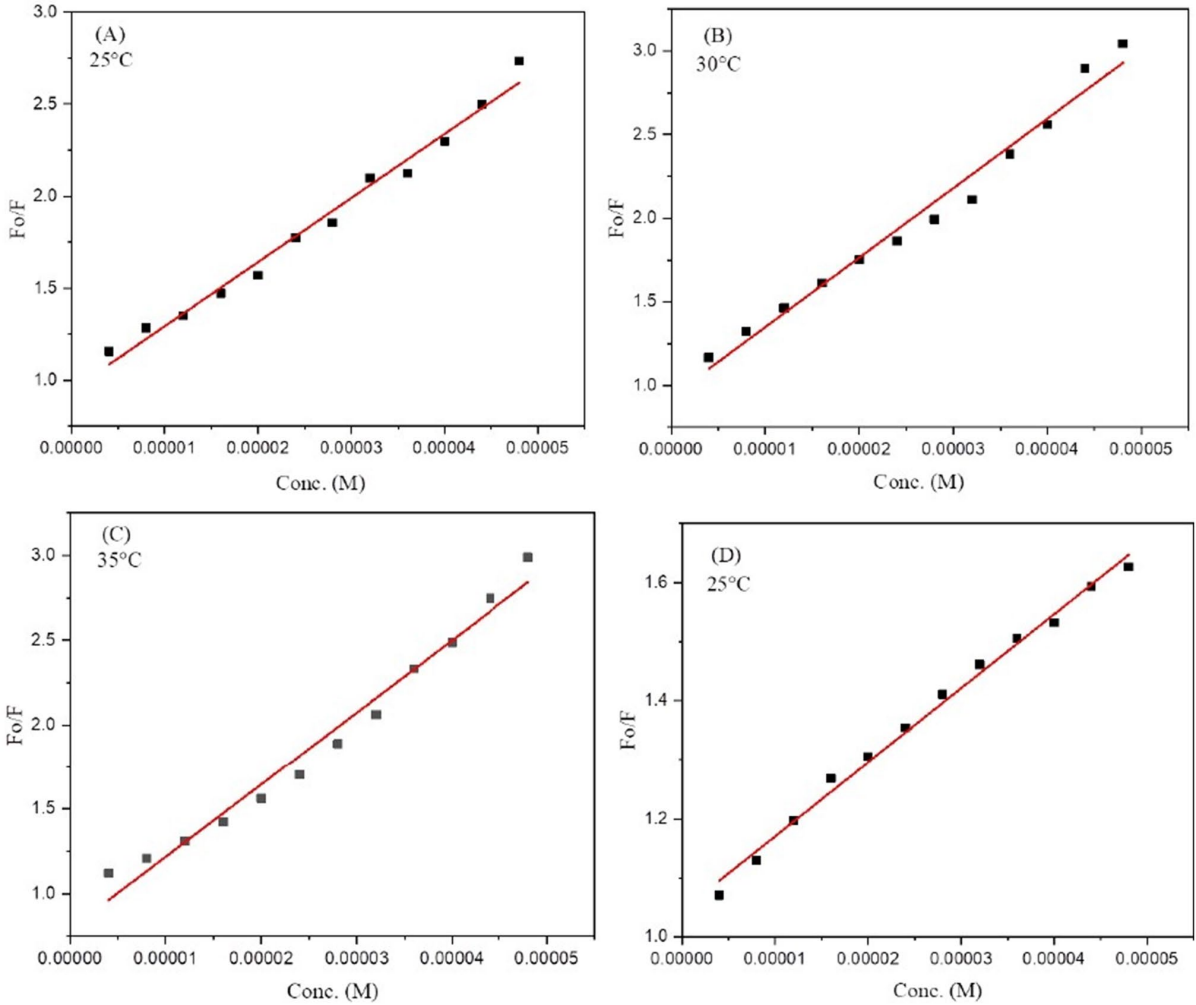
Stern–Volmer plots for the DNA/BSA-**4g** complex system (**A**) BSA-4g complex at 25 °C; (**B**) BSA-4g complex 30 °C; (**C**) BSA-4g complex at 35 °C; (**D**) DNA-4g complex at 25 °C.

To detect how effectively ligand **4g** binds with DNA/BSA, fluorescent data were utilized to compute quenching constant (*Kq*) and Stern–Volmer constant (*Ksv*) by plotting emission spectral data at λ_max._ (342 nm) at three different temperatures (25 °C, 30 °C, 35 °C) with BSA and λ_max._ (314 nm) at 25 °C with DNA by employing the Stern–Volmer equation (Eq. 1) In each case a linear fit graph was obtained when the ratio of fluorescence intensity (F_o_) of DNA in the absence of ligand to the fluorescence intensity (F) of DNA in the presence of ligand **4g** at varying concentration was plotted against increasing concentration of ligand **4g** (Fig. 11). The results obtained for the quenching constant (*Kq*) and Stern–Volmer constant (*Ksv*) are portrayed in Table 6. The decrease in the value of *Kq* with an increase in temperature indicates that rate of quenching diminished at higher temperatures in the case of BSA. The value of quenching constant *Kq* was found to be higher as compared to scattering constant^40^ (10^10^ L/mol s) signifying the complex formation between quencher **4g** and fluorophore (protein) and thus suggesting the static mechanism to be followed in both cases.

**Table 6:** Stern–Volmer constant (*Ksv*), Binding constant (*K*_*b*_), number of binding sites (*n*) and Quenching constant (*Kq*).

DNA/BSA binding	T (°C)	*Ksv* (M^−1^)	*Kq* (M^−1^ s^−1^)	Log K_b_	*K*_*b*_ (M^−1^)	*n*	∆G° (kcal/mol)
BSA binding	25	(4.2 ± 0.2) × 10^4^	(4.2 ± 0.2) × 10^12^	5.2 ± 0.2	1.8 × 10^5^	1.1 ± 0.04	− 7.06
	30	(4.1 ± 0.1) × 10^4^	(4.1 ± 0.1) × 10^12^	4.5 ± 0.1	3.3 × 10^4^	0.9 ± 0.02	− 6.21
	35	(3.4 ± 0.1) × 10^4^	(3.4 ± 0.1) × 10^12^	4.3 ± 0.1	2.2 × 10^4^	0.9 ± 0.03	− 5.94
DNA binding	25	(1.2 ± 0.03) × 10^4^	(1.2 ± 0.03) × 10^12^	3.6 ± 0.06	3.9 × 10^3^	0.8 ± 0.01	− 4.8

Binding constant ***K***_***b***_ and number of binding sites (n) were also calculated using modified form of Stern–Volmer equation^41^ (Eq. 2).

2 $$\mathrm{Log}\frac{(Fo-F)}{F} = n\mathrm{Log}\left[\mathrm{Q}\right]+\mathrm{ Log }K_b.$$

**Figure 12:**
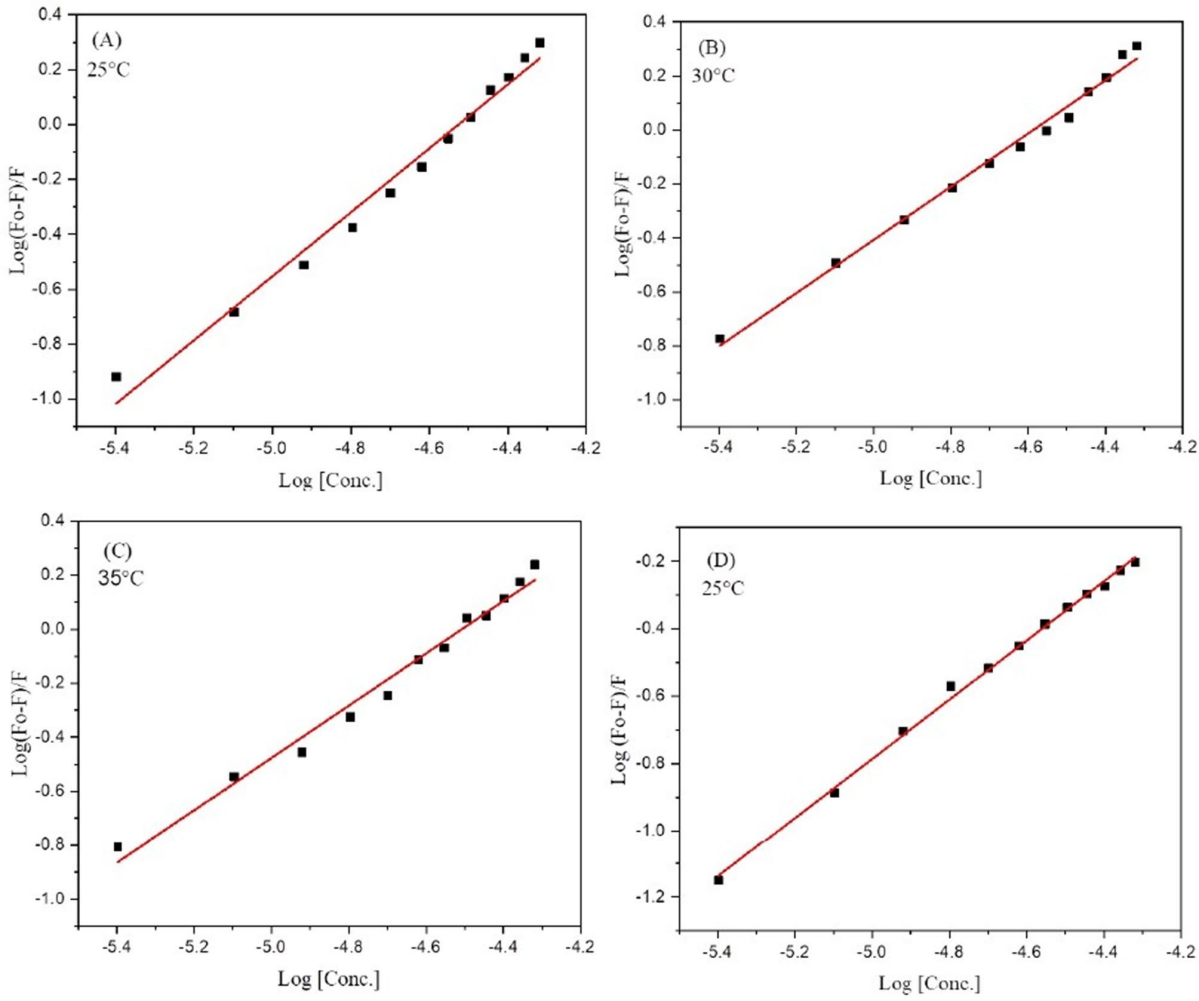
Modified Stern–Volmer plots for the DNA/BSA-**4g** complex system (**A**) BSA-4g complex at 25 °C; (**B**) BSA-4g complex 30 °C; (**C**) BSA-4g complex at 35 °C; (**D**) DNA-4g complex at 25 °C.

The fluorescent data were further utilized to determine how strongly ligand binds with DNA using a modified Stern–Volmer equation (Eq. 2). When the logarithmic ratio of fluorescent data was plotted against the logarithmic concentration of ligand **4g** a straight-line graph was obtained (Fig. 12). The binding constant *K*_*b*_ obtained from the intercept whereas the number of binding sites n were easily calculated from slope of the plot and are mentioned in Table 6 indicating moderate binding of ligand **4g** with DNA/BSA. From the results, it is easily concluded that the affinity of binding decreases with the rise in temperature in case of BSA.

Furthermore, the spontaneity of the binding process was examined by calculating the change in standard Gibbs free energy change (∆G°) by employing the following equation.

$$\Delta {\text{G}}^{^\circ } = \, - {\text{RTln}}K_{b} .$$

The negative values of ∆G° in each case are demonstrated in Table 6. The negative value of ∆G° indicates that the binding process between ligand **4g** and DNA/BSA is spontaneous.

### Competitive displacement assay

**Figure 13:**
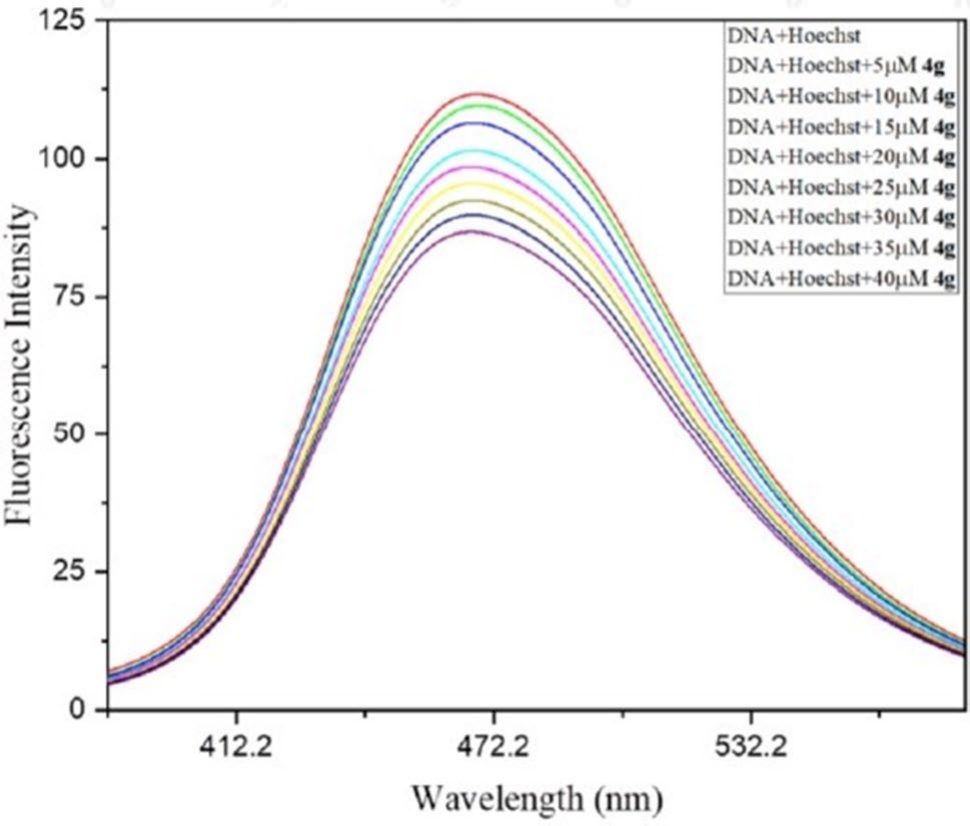
Competitive displacement plot of DNA–Hoechst complex in the absence and presence of varying amount of **4g** (0–40 µM).

Competitive displacement assay is a powerful tool to identify the mode of binding between DNA and ligand (groove binder or intercalator). Ethidium bromide (EtBr) is a well-known intercalator DNA dye, since it binds with the double-stranded DNA by residing in the base pairs of DNA^42^ and the Hoechst 33258 dye binds strongly with DNA through groove binding mode^43^. In Fluorescent displacement assay, DNA–EtBr complex was excited at 471 nm and DNA–Hoechst complex was excited at 343 nm. The emission spectra of DNA–EtBr and DNA–Hoechst complexes were recorded from 500 to 700 nm and 360–600 nm in the absence and presence of increasing concentration of ligand **4g,** respectively. Any modification in the fluorescence spectra of the respective dye complexes (DNA–EtBr or DNA–Hoechst complexes) on sequential addition of **4g** will suggest the corresponding mode of bindings between DNA and ligand. In fluorescence spectra, there is a gradual decrease in the emission intensity of DNA–Hoechst complex was observed (Fig. 13), however in spectrum of DNA–EtBr complex, there is no change in the emmision peak even at high concentration of **4g** (Supplementary Fig. S3). This suggests that compound **4g** interacts with DNA through groove binding modes and ruled out the possibility of **4g** to be an intercalator.

### Circular dichroism

CD spectrum of DNA comprises two characteristic bands; a positive band at 275 nm due to π–π base stacking and a negative band at 245 nm due to helicity of B form DNA^44^. Intercalation of small molecules with DNA enhances the intensities of both the bands, however, groove binding shows little or no perturbations on the base stacking and helicity bands, stabilizing the right-handed helical structure of ct-DNA. Herein, CD spectral variations of ct-DNA were recorded by the sequential addition of the ligand **4g** to ct-DNA (Supplementary Fig. S4). Outcomes of this study showed that intensities of both the bands do not alter significantly. This suggests that thiazolopymidine derivatives bind to ct-DNA through a non-intercalative mode of binding and offer support to their groove binding nature. Results of CD spectra are in very well supported the absorptive, quenching, molecular docking.

### Viscosity experiment

Viscosity studies were conducted to confirm, whether the ligand **4g** binds with DNA as a groove binder or as an intercalator. For the whole procedure, an Ubbelohde viscometer was used to carry out the viscometric measurements at 25 °C (± 0.01) by hanging vertically into a thermostat to keep the temperature constant. Firstly, the viscosity of pure DNA (100 µM) was recorded in the absence of ligand **4g** and then the viscosity of DNA in the presence of an increasing concentration of compound **4g** was recorded. The flow time was measured using a digital stopwatch and each sample was tested three times to get accurate outcomes. The relative viscosity was determined using the following relation^45^

$$\frac{\eta }{{\eta }_{o}} = \frac{({t}_{complex }- {t}_{o})/{t}_{o}}{({t}_{DNA}-{t}_{o})/ {t}_{o}},$$

where ƞ and ƞ_o_ defines the viscosities of DNA in the presence and absence of **4g** respectively, t_DNA_ is the average flow time of pure DNA, t_complex_ is the average flow time of DNA in the presence of **4g** at varying concentration, and t_o_ be the flow time of Tris–HCl buffer^46^. If a ligand binds via intercalary mode an increase in viscosity will be observed however, no effect on viscosity will be detected in case of groove binders^47^.

**Figure 14:**
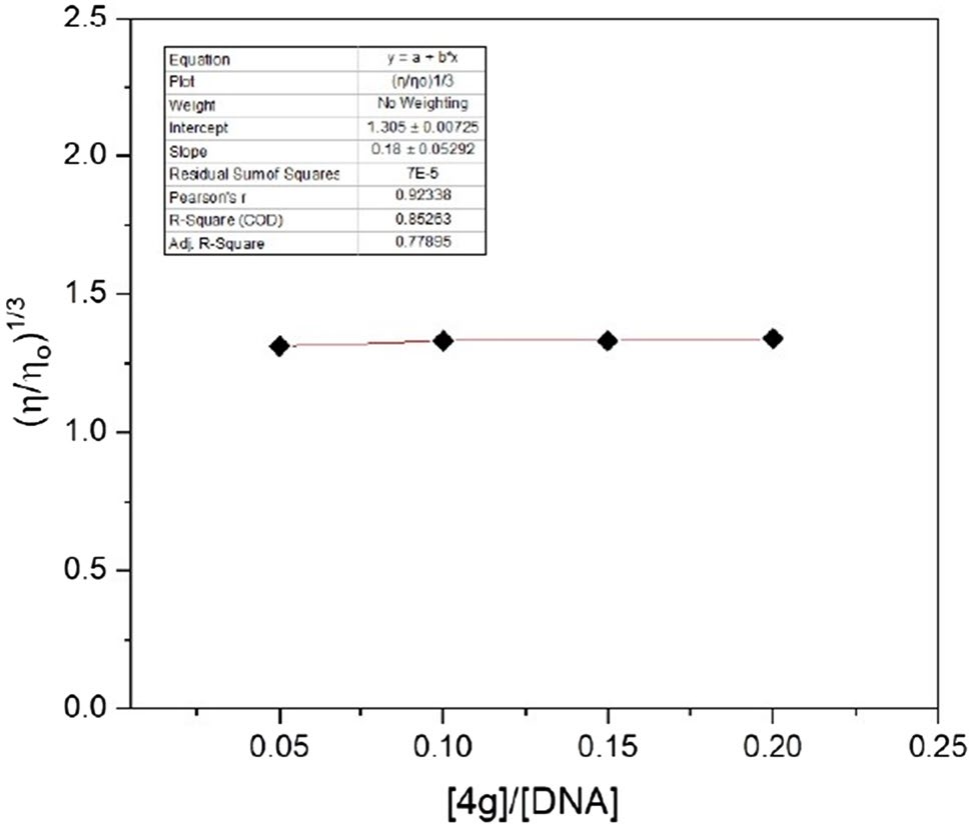
Effect on relative viscosity of DNA of fixed concentration by increasing the concentration of ligand **4g**.

The results thus obtained using the above equation were used and a straight-line graph parallel to the concentration axis was obtained when the ratio of the concentration of **4g** to DNA ([4g]/[DNA]) was plotted against (ƞ/ƞ_o_)^1/3^ (Fig. 14). It is very clear from the results that, negligible change in the relative viscosity was noticed on continuously increasing the concentration of ligand **4g** indicating that the ligand **4g** will behave as a groove binder rather than an intercalator. These results are in favor of the outcomes extracted from the competitive displacement assay and CD studies.

## Conclusion

In conclusion, regioselective synthesis of a series of 2-aroyl/heteroaroyl-3-methyl-6,7-dihydro-5*H*-thiazolo[3,2-*a*]pyrimidines **4a–h** out of the four possible regioisomers has been accomplished using unsymmetrical 1,3-diketones **1a–h** and tetrahydropyrimidine-2-(*H*)-thione **2** as simple synthons in presence of visible light conditions and the products are obtained in excellent yields. The synthesized compounds have been characterized by IR, ^1^H and ^13^C NMR and 2D-NMR [(^1^H-^13^C) HSQC and (^1^H-^13^C) HMBC)]. In silico toxicity analysis revealed that synthesized derivatives are having less toxicity risks to Mutagenicity, Tumorigenicity, Irritancy and Reproductive effects with remarkable drug score. Molecular docking studies displayed that among the synthesized thiazolopyrimidines compound with 2,4-dichloro substitution **4g** interacts with BSA protein through non-covalent forces in the active pocket region of chain A, however, these compounds interact with DNA in the Guanine-Cytosine rich region in the minor groove. Further biophysical studies evidenced that thiazolopyrimidine derivative binds with DNA/BSA more efficiently. The binding constant value obtained from fluorescence quenching studies specifies that compound **4g** binds moderately with BSA protein through static quenching. The molecule synthesized shows a moderate binding with DNA and BSA and thus can be further evaluated as DNA-binding chemotherapeutic agent in cancer cells. Additionally, the outcomes of this work motivate the researchers to design, develop and synthesize novel thiazolopyrimidine derivatives with remarkable biochemical properties.

### Experimental

#### Chemistry

*General methods* Melting points were determined in open capillaries on digital Melting Point Apparatus (MEPA) and were uncorrected. Analytical TLC was performed using Merck Kieselgel 60 F254 silica gel plates. Visualization was performed by UV light (254 nm). The Visible light source used was of power 27 W and was placed 15 cm away from the reaction medium. IR spectra were recorded on Buck Scientific IR M-500 spectrophotometer using KBr pellets (υ_max_ in cm^−1^), ^1^H and ^13^C NMR spectra on a Bruker instrument at 500 and 125 MHz respectively, using deuteriochloroform as a solvent and the chemical shifts are expressed in δ-scale downfield from TMS as internal standard. Elemental analyses were performed at Sophisticated Analytical Instrumentation Facility, Panjab University, Chandigarh. All the compounds gave C, H and N analysis within ± 0.5 of the theoretical values. High-resolution mass spectra (HRMS) were measured in ESI^+^ mode at MRC, MNIT, Jaipur. 2D correlation spectra, (^1^H-^13^C) gs-HMQC and (^1^H-^13^C) gs-HMBC of samples **4a** were carried out at IIT, Mandi.

### General method of synthesis

1,3-Diketones^18^ and tetrahydropyrimidine-2-thione were prepared according to the literature procedure. NBS was purchased commercially.

### General method for preparation of 2-aroyl/heteroaroyl-3-methyl-6,7-dihydro-5*H*-thiazolo[3,2-*a*]pyrimidines (4a–h)

1,3-Diketone (1.0 eq) and NBS (0.177 g, 1.0 eq) were taken in dry mortar and grounded using a pestle until a thick paste was formed. The thick paste so formed was eventually added with tetrahydropyrimidine-2-thione (0.116 g, 0.1 eq) in a 4:1 solution of ethanol and water in a conical flask and the reaction was progressed in presence of a visible light source of 27 W at room temperature. On completion of the reaction as indicated by TLC, the volume of the solvent was reduced using a rotatory evaporator and the solid so obtained was filtered, recrystallized with ethanol and dried to get **4a–h** in 78–88% yields.

#### 2-Benzoyl-3-methyl-6,7-dihydro-5H-thiazolo[3,2-a]pyrimidine (4a)

Brown crystals; M. Pt. 125 °C; Yield: 85%; IR (KBr) ν_max_ (cm^−1^): 1587 (C=O); ^1^H NMR (500 MHz, CDCl_3_) δ: 7.68–7.67 (d, 2H, ^*3*^*J* = 7.5 Hz, 2′,6′-H), 7.53–7.50 (t, 1H, ^*3*^*J* = 7.0 Hz, ^3^*J* = 7.5 Hz, 4′-H), 7.45–7.42 (t, 2H, ^*3*^*J* = 8.0 Hz, ^3^*J* = 7.5 Hz, 3′,5′-H), 3.77–3.75 (t, 2H, ^*3*^*J* = 6.0 Hz, ^3^*J* = 6.0 Hz, 5-H), 3.53–3.51 (t, 2H, ^*3*^*J* = 5.5 Hz, ^3^*J* = 5.5 Hz, 7-H), 2.25 (s, 3H, 3-CH_3_), 2.00–1.95 (quintet, 2H, ^*3*^*J* = 6.0 Hz, ^3^*J* = 5.5 Hz, ^*3*^*J* = 6.0 Hz, ^3^*J* = 6.0 Hz, 6-H); ^13^C NMR (125 MHz, CDCl_3_) δ: 187.9, 157.6, 146.4, 140.2, 131.9, 128.5, 128.1, 109.1, 45.3, 43.1, 19.9, 13.9; HRMS (ESI): *m/z* calcd for C_14_H_14_N_2_OS: 258.0827; found: 259.0905 [M + 1]^+^; Elemental analysis: Calcd. for C_14_H_14_N_2_OS: C, 65.09; H, 5.46; N, 10.84% Found: C, 65.04; H, 5.42; N, 10.82%.

#### 2-(4-Methyl benzoyl)-3-methyl-6,7-dihydro-5H-thiazolo[3,2-a]pyrimidine (4b)

Buff coloured solid; M.Pt. 146.5 °C; Yield 82%; IR (KBr) ν_max_ (cm^−1^): 1593 (C=O); ^1^H NMR (500 MHz, CDCl_3_) δ: 7.58–7.56 (d, 2H, ^*3*^*J* = 8.0 Hz, 2′,6′-H), 7.21–7.20 (d, 1H, ^*3*^*J* = 8.5 Hz, 3′,5′-H), 3.77–3.75 (t, 2H, ^*3*^*J* = 5.5 Hz, ^3^*J* = 6.0 Hz, 5-H), 3.51–3.49 (t, 2H, ^*3*^*J* = 5.5 Hz, ^3^*J* = 5.0 Hz, 7-H), 2.38 (s, 3H, 4′-CH_3_), 2.24 (s, 3H, 3-CH_3_), 1.98–1.95 (quintet, 2H, ^*3*^*J* = 6.0 Hz, ^3^*J* = 5.5 Hz, ^*3*^*J* = 6.0 Hz, ^3^*J* = 6.0 Hz, 6-H); ^13^C NMR (125 MHz, CDCl_3_) δ: 187.6, 158.5, 145.6, 142.8, 137.1, 130.2, 129.7, 129.2, 128.4, 109.8, 44.6, 43.1, 21.7, 19.7, 13.8; HRMS (ESI): *m/z* calcd for C_15_H_16_N_2_OS: 272.0983; found: 273.1060 [M + 1]^+^; Elemental analysis: Calcd. for C_15_H_16_N_2_OS: C, 66.15; H, 5.92; N, 10.29% Found: C, 66.10; H, 5.88; N, 10.26%.

#### 2-(4-Bromobenzoyl)-3-methyl-6,7-dihydro-5H-thiazolo[3,2-a]pyrimidine (4c)

Creamy white solid; M. Pt. 153.5 °C; Yield: 88%; IR (KBr) ν_max_ (cm^−1^): 1585 (C=O); ^1^H NMR (500 MHz, CDCl_3_) δ: 7.57 (s, 4H, 2′,3′,5′,6′-H), 3.78–3.76 (t, 2H, ^*3*^*J* = 6.0 Hz, ^3^*J* = 6.0 Hz, 5-H), 3.53–3.51 (t, 2H, ^*3*^*J* = 5.5 Hz, ^3^*J* = 6.0 Hz, 7-H), 2.30 (s, 3H, 3-CH_3_), 2.01–1.96 (quintet, 2H, ^*3*^*J* = 5.5 Hz, ^3^*J* = 6.0 Hz, ^*3*^*J* = 5.5 Hz, ^3^*J* = 6.0 Hz, 6-H); ^13^C NMR (125 MHz, CDCl_3_) δ: 186.5, 146.8, 138.7, 131.7, 129.7, 126.6, 45.1, 43.0, 19.7, 13.8; HRMS (ESI): *m/z* calcd for C_14_H_13_BrN_2_OS: 335.9932; found: 337.0015 [M + 1]^+^; 338.9980 [M + 1 + 2]^+^, (1:1); Elemental analysis: Calcd. for C_14_H_13_BrN_2_OS: C, 49.86; H, 3.89; N, 8.31% Found: C, 49.81; H, 3.85; N, 8.29%.

#### 2-(4-Fluorobenzoyl)-3-methyl-6,7-dihydro-5H-thiazolo[3,2-a]pyrimidine (4d)

Creamy white solid; M. Pt. 149 °C; Yield: 83%; IR (KBr) ν_max_ (cm^−1^): 1585 (C=O); ^1^H NMR (500 MHz, CDCl_3_) δ: 7.75–7.71 (q, 2H, ^*3*^*J* = 8.5 Hz, ^*3*^*J* = 9.0 Hz, 2′,6′-H), 7.13–7.09 (t, 2H, ^*3*^*J* = 11.0 Hz, ^3^*J* = 10.5 Hz, 3′,5′-H), 3.79–3.76 (t, 2H, ^*3*^*J* = 7.5 Hz, ^3^*J* = 7.0 Hz, 5-H), 3.53–3.51 (t, 2H, ^*3*^*J* = 7.0 Hz, ^3^*J* = 7.0 Hz, 7-H), 2.30 (s, 3H, 3-CH_3_), 2.01–1.95 (quintet, 2H, ^*3*^*J* = 7.0 Hz, ^3^*J* = 7.5 Hz, ^*3*^*J* = 7.0 Hz, ^3^*J* = 7.0 Hz, 6-H); ^13^C NMR (125 MHz, CDCl_3_) δ: 186.3, 166.1, 163.6, 157.3, 146.5, 136.2, 130.6, 115.6, 115.4, 108.1, 45.2, 42.9, 19.8, 13.7; HRMS (ESI): *m/z* calcd for C_14_H_13_FN_2_OS: 276.0733; found: 277.0812 [M + 1]^+^; Elemental analysis: Calcd. for C_14_H_13_FN_2_OS: C, 60.85; H, 4.74; N, 6.88% Found: C, 60.80; H, 4.70; N, 6.85%.

#### 2-(4-Methoxybenzoyl)-3-methyl-6,7-dihydro-5H-thiazolo[3,2-a]pyrimidine (4e)

Brown solid; M. Pt. 141.5 °C; Yield: 82%; IR (KBr) ν_max_ (cm^−1^): 1589 (C=O); ^1^H NMR (500 MHz, CDCl_3_) δ: 7.7 (d, 2H, ^3^*J* = 8.8 Hz, 2′,6′-H), 6.92–6.91 (d, 2H, ^3^*J* = 8.8 Hz, 3′,5′-H), 3.86 (s, 3H, 4′-OCH_3_), 3.77–3.74 (t, 2H, ^3^*J* = 5.9 Hz*,* ^3^*J* = 5.9 Hz, 5-H), 3.52–3.50 (t, 2H, ^3^*J* = 5.5 Hz, ^3^*J* = 5.6 Hz*,* 7-H), 2.27 (s, 3H, 3-CH_3_), 1.99–1.94 (quintet, 2H, ^3^*J* = 5.7 Hz, ^3^*J* = 5.7 Hz, ^3^*J* = 5.8 Hz, ^3^*J* = 5.8 Hz, 6-H); ^13^C NMR (125 MHz, CDCl_3_) δ: 186.7, 162.7, 157.7, 145.4, 132.5, 130.6, 113.6, 108.5, 55.5, 45.3, 42.9, 19.9, 13.8; HRMS (ESI): *m/z* calcd for C_15_H_16_N_2_O_2_S: 288.0966; found: 289.1017 [M + 1]^+^; Elemental analysis: Calcd. for C_15_H_16_N_2_O_2_S: C, 62.47; H, 5.55; N, 9.71% Found: C, 62.42; H, 5.51; N, 9.68%.

#### 2-(2-Methoxybenzoyl)-3-methyl-6,7-dihydro-5H-thiazolo[3,2-a]pyrimidine (4f)

Red solid; M. Pt. 148.5 °C; Yield: 81%; IR (KBr) ν_max_ (cm^−1^): 1585 (C=O); ^1^H NMR (500 MHz, CDCl_3_) δ: 7.42–7.38 (m, 1H, 6′-H), 7.24–7.22 (dd, 1H, ^3^*J* = 7.4 Hz, ^5^*J* = 7.4 Hz, 4′-H), 7.00–6.97 (m, 1H, 5′-H), 6.95–6.93 (d, ^3^*J* = 8.3 Hz, 1H, 3′-H), 3.81 (s, 3H, 2′-OCH_3_), 3.73–3.70 (t, 2H, ^3^*J* = 5.9 Hz, ^3^*J* = 5.9 Hz, 5-H), 3.49–3.47 (t, 2H, ^3^*J* = 5.5 Hz, ^3^*J* = 5.5 Hz, 7-H), 2.09 (s, 3H, 3-CH_3_), 1.97–1.93 (quintet, 2H, ^3^*J* = 5.9 Hz, ^3^*J* = 5.6 Hz, ^3^*J* = 5.6 Hz, ^3^*J* = 5.7 Hz, 6-H); ^13^C NMR (125 MHz, CDCl_3_) δ: 186.7, 157.6, 156.1, 146.0, 131.5, 130.4, 128.1, 120.8, 112.2, 111.5, 55.8, 45.1, 43.1, 19.8, 12.9; HRMS (ESI): *m/z* calcd for C_15_H_16_N_2_O_2_S: 288.0966; found: 289.1017 [M + 1]^+^; Elemental analysis: Calcd. for C_15_H_16_N_2_O_2_S: C, 62.47; H, 5.55; N, 9.71% Found: C, 62.43; H, 5.49; N, 9.68%.

#### 2-(2,4-Dichlorobenzoyl)-3-methyl-6,7-dihydro-5H-thiazolo[3,2-a]pyrimidine (4g)

Yellow solid; M. Pt. 224.5 °C; Yield: 84%; IR (KBr) ν_max_ (cm^−1^): 1595 (C=O); ^1^H NMR (500 MHz, CDCl_3_) δ: 7.45 (d, 1H, ^3^*J* = 2.1 Hz, 3′-H), 7.33–7.31 (dd, 1H, ^3^J = 8.2 Hz, ^5^*J* = 8.3 Hz, 5′-H), 7.26–7.25 (d, 1H, ^3^*J* = 8.2 Hz, 6′-H), 3.75–3.73 (t, 2H, ^3^*J* = 6.3, ^3^*J* = 6.3, 5-H), 3.52–3.49 (t, 2H, ^3^*J* = 5.9, ^3^*J* = 5.9, 7-H), 2.10 (s, 3H, 3-CH_3_), 1.99–1.94 (quintet, 2H, ^3^*J* = 5.9 Hz, ^3^*J* = 6.2 Hz, ^3^*J* = 6.2 Hz, ^3^*J* = 5.7 Hz, 6-H); ^13^C NMR (125 MHz, CDCl_3_) δ: 184.1, 156.7, 147.5, 138.3, 136.2, 131.5, 130.1, 128.8, 127.6, 111.0, 45.4, 43.2, 19.9, 13.0; HRMS (ESI): *m/z* calcd for C_14_H_12_Cl_2_N_2_OS: 326.0047; found: 327.0048 [M + 1]^+^, 329.0009 [M + 1 + 2]^+^, 330.9972 [M + 1 + 4]^+^ (9:6:1); Elemental analysis: Calcd. for C_14_H_12_Cl_2_N_2_OS: C, 51.53; H, 3.68; N, 8.58% Found: C, 51.49; H, 3.65; N, 8.54%.

#### 2-Thienoyl-3-methyl-6,7-dihydro-5H-thiazolo[3,2-a]pyrimidine (4h)

Dark Brown solid; M. Pt. 223.5 °C; Yield: 78%; IR (KBr) ν_max_ (cm^−1^): 1571 (C=O); ^1^H NMR (500 MHz, CDCl_3_) δ: 7.45–7.44 (m, 1H, 3′-H), 7.34–7.30 (m, 1H, 5′-H), 7.27 (s, 1H, 4′-H), 3.76–3.73 (t, 2H, ^3^*J* = 7.5 Hz*,* ^3^*J* = 7.5 Hz*,*5-H), 3.52–3.50 (t, 2H, ^3^*J* = 6.8 Hz*,* ^3^*J* = 7.2 Hz*,*7-H), 2.12 (s, 3H, 3-CH_3_), 2.0–1.94 (quintet, 2H, ^3^*J* = 7.3 Hz, ^3^*J* = 7.1 Hz, ^3^*J* = 7.2 Hz, ^3^*J* = 7.2 Hz, 6-H); ^13^C NMR (125 MHz, CDCl_3_) δ: 184.1, 147.4, 138.4, 136.4, 131.6, 130.1, 128.9, 127.6, 45.1, 43.2, 19.7, 13.0; HRMS (ESI): *m/z* calcd for C_12_H_12_N_2_OS_2_: 264.0391; found: 265.0391 [M + 1]^+^; Elemental analysis: Calcd. for C_12_H_12_N_2_OS_2_: C, 54.53; H, 4.54; N, 10.60% Found: C, 54.46; H, 4.51; N, 10.58%.

#### Molecular docking studies

Structures of ligands were drawn in ChemDraw Professional 15.0 software and crystal structure of the Bovine serum albumin BSA protein (PDB ID; 4f5s) and B-DNA dodecamer (PDB ID; 1BNA) were obtained from Protein Data Bank (https://www.rcsb.org/pdb). Protein file rectify by removing water molecules, small residues like triethylene Glycol (PGE) and by packing with polar hydrogen with Kollman charges using MGL Tools program. DNA receptor file was also fixed by eliminating water molecules and adding polar hydrogen. Blind molecular docking was performed using AutoDock Vina software including a Lamarckian genetic algorithm for calculations. The output results were analyzed in BIOVIA Discovery Studio Visualizer (DSV) and the lowest energy pose of the thiazolopyrimidine derivatives was considered as the best binding mode for the most stable drug-receptor complex.

### Material and instrumentation

Calf-thymus DNA (ct-DNA) of molecular biology ranking (fibers form), Bovine serum albumin and ethidium bromide (EtBr) were purchased directly from Sigma Aldrich Company and exploited without any absolution. Hoechst 33258 dye was procured from HiMedia. For the whole interaction studies, analytical grade reagents were used.

For fluorescence, spectral analysis xenon lamp reinforced Hitachi F-4700 quantum north-west 5J204700 instrument was employed using 10 mm path length quartz cuvette. UV–visible spectral analysis was achieved by xenon lamp equipped Thermo Scientific’s Evolution 300 spectrophotometer using conventional quartz cell of 10 mm path length. CD spectra (Far-UV, 200–250 nm) DNA-**4g** complex system were recorded on J-815spectrophotometer (JASCO, Japan) at room temperature using a quartz cuvette with a path length of 10 mm. CD spectrometer was calibrated using Camphorsulfonic acid.

### Stock solution preparation

For the preparation of a stock solution of (2,4-dichlorophenyl)(3-methyl-6,7-dihydro-5*H*-thiazolo[3,2-*a*]pyrimidin-2-yl) methanone **(4g)** having 4 mM concentration, DMSO was used as a solvent and further diluted as per requirement depending upon the mode of interaction study. BSA stock solution of 15 µM (1 mg/1 ml) concentration was prepared using Phosphate Buffer saline (prepared using Na_2_HPO_4_ and NaH_2_PO_4_) of 10 mM concentration with pH 7.4.

ct-DNA was homogenized by suspending in 10 mM Tris–HCl buffer (pH 7.2, containing 0.1 M HCl) with occasional mixing utilizing vortex. Beer-Lambert’s Law (A = εlc) was employed to determine the precise concentration of DNA at 260 nm which was found to be 72 µM using molar extinction coefficient (6600 M^−1^ cm^−1^) for an isolated strand of ct-DNA. The attenuance ratio^48^ A_260_/A_280_ was found to be in between 1.8 to1.9 using absorption spectroscopy indicative of protein free DNA and thus there was no need of any kind of DNA purification.

## Supplementary Information

Supplementary Information.
