# Supplementary material for: Visible-light driven regioselective synthesis, characterization and binding studies of 2-aroyl-3-methyl-6,7-dihydro-5H-thiazolo[3,2-a]pyrimidines with DNA and BSA using biophysical and computational techniques
Source: Sci Rep. 2021 Nov 11;11:22135. doi: 10.1038/s41598-021-01037-4 (PMC8586366; doi:10.1038/s41598-021-01037-4)
Supplement: Supplementary file 1 — Supplementary Information. [file 41598_2021_1037_MOESM1_ESM.docx]

**SUPPLEMENTARY DATA**

**For**

**Visible-light Driven Regioselective Synthesis, Characterization and Binding Studies of 2-Aroyl-3-methyl-6,7-dihydro-5*H*-thiazolo[3,2-*a*]pyrimidines with DNA and BSA Using Biophysical and Computational techniques**

Ranjana Aggarwal,^a,b^* Naman Jain,^a^ Shilpa Sharma,^a^ Prince Kumar,^a^ Gyan Prakash Dubey,^a^ Heerak Chugh,^c^ Ramesh Chandra,^c^

^a^Department of Chemistry, Kurukshetra University, Kurukshetra-136119, Haryana, India

^b^CSIR-National Institute of Science Communication and Policy Research, New Delhi 110012, India

^c^Department of Chemistry, University of Delhi, New Delhi 110007, India

*Corresponding author:

Prof. Ranjana Aggarwal, CSIR- National Institute of Science Communication and Policy Research, New Delhi 110012, India. Tel: +91-9896740740

E-mails: [*ranjana67in@yahoo.com*](mailto:ranjana67in@yahoo.com), [*ranjanaaggarwal67@gmail.com*](mailto:ranjanaaggarwal67@gmail.com)

**TABLE of CONTENT**

| Absorption spectra of ctDNA/BSA  **Figure S1, S2** | Page no. |
| --- | --- |
|  | S3 |
| Ethidium Bromide displacement assay  **Figure S3** | S4 |
| CD spectra of ct-DNA  **Figure S4** | S4 |
| **Characterization of Final Compounds**  ^1^H NMR, ^13^C NMR, HMBC, HMQC  **Figures S5-S15, Tables S1-S3** | S5-S18 |
| HRMS spectra  **Figures S16-S22** | S19-S22 |


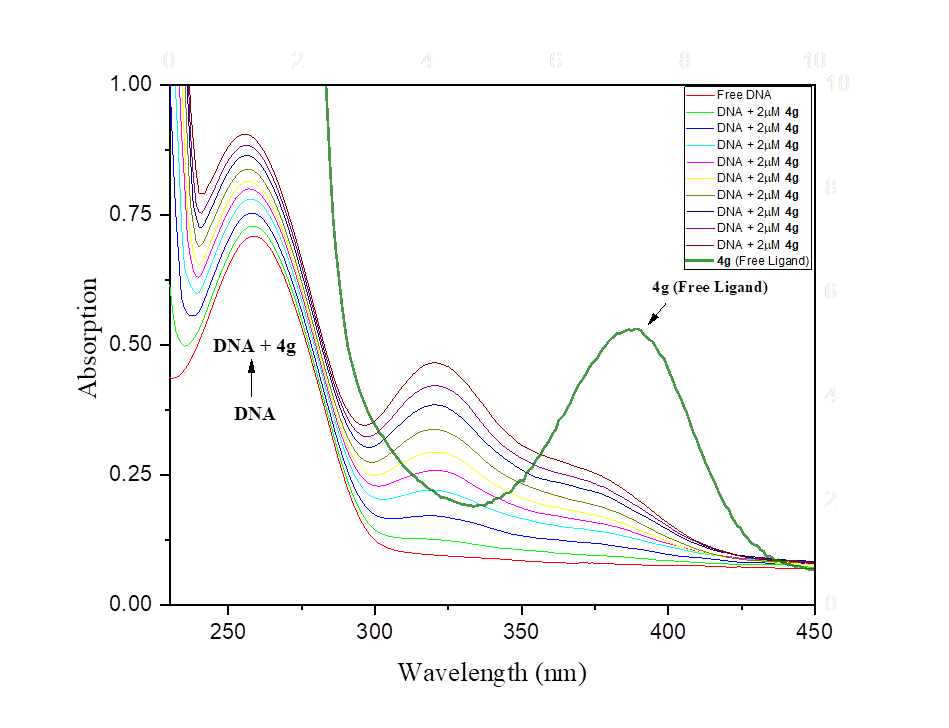


**Figure S1.** Absorption spectra of ctDNA at the increasing concentration of **4g** while correcting the baseline after each addition.


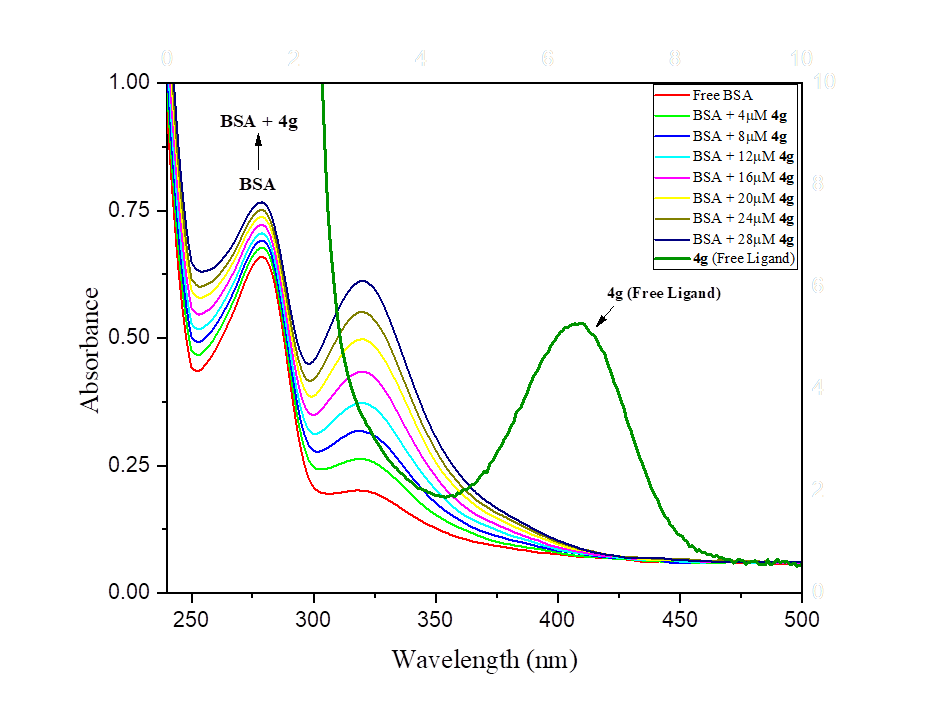


**Figure S2.** Absorption spectra of BSA at the increasing concentration of **4g** while correcting the baseline after each addition.

**
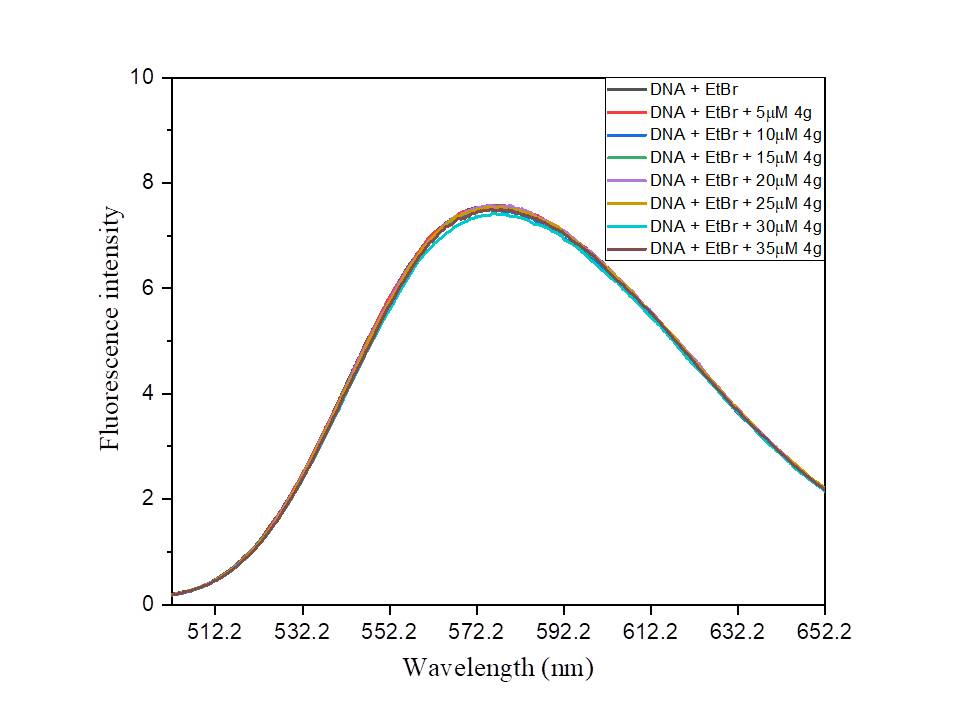
**

**Figure S3**. Fluorescent intercalator displacement assay plot of DNA-EtBr complex in the absence and presence of varying amount of **4g** (0-35µM).


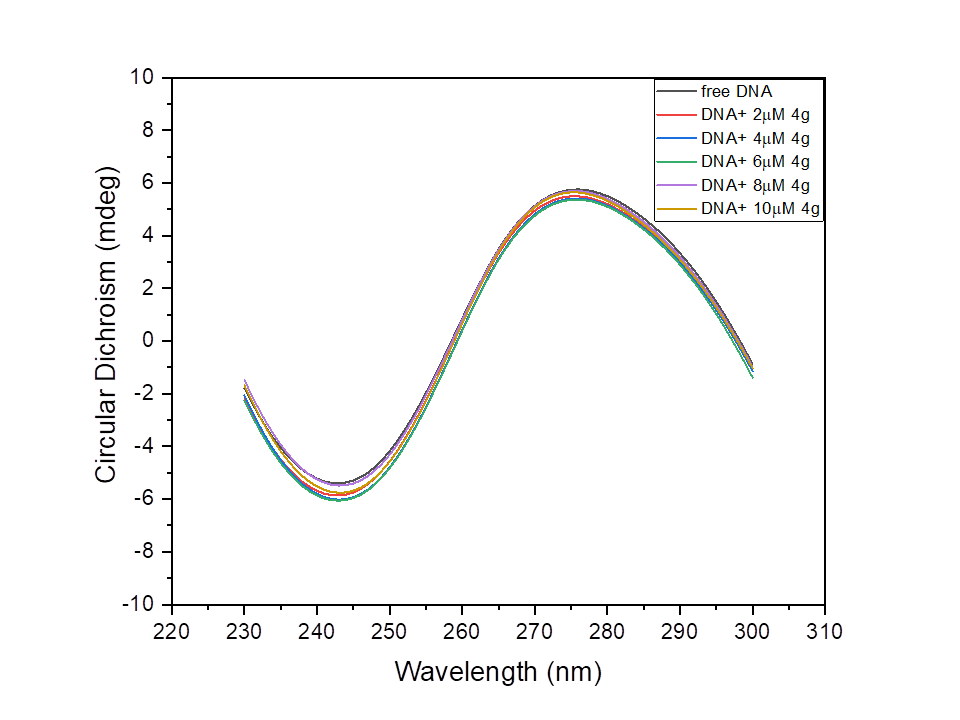


**Figure S4.** CD spectra of ct-DNA with varying concentrations of **4g**.

**Characterization of Final Compounds**

**^1^H NMR, ^13^C NMR, HMQC, HMBC of Final Compounds**

1. 2-benzoyl-3-methyl-6,7-dihydro-5*H*-thiazolo[3,2-*a*]pyrimidine (**4a**)

**
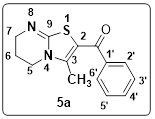
**

**Figure S5a.** ^1^H NMR spectrum of **4a
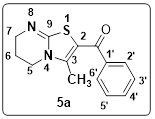
**

**Figure S5b.** ^13^C NMR spectrum of **4a**

1. **
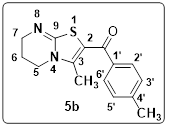
**2-(4-methyl benzoyl)-3-methyl-6,7-dihydro-5*H*-thiazolo[3,2-*a*]pyrimidine (**4b**)

**Figure S6a.**^1^H NMR spectrum of **4b**

**
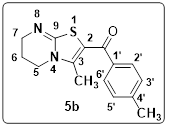
**

**Figure S6b.** ^13^C NMR spectrum of **4b**

1. **
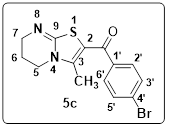
**^^2-(4-bromobenzoyl)-3-methyl-6,7-dihydro-5*H*-thiazolo[3,2-*a*]pyrimidine (**4c**)

**Figure S7a.** ^1^H NMR spectrum of **4c**

**
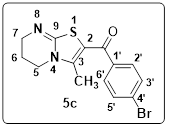
**

**Figure S7b.** ^13^C NMR spectrum of **4c**

1. **
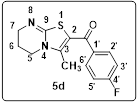
**^^2-(4-fluoro benzoyl)-3-methyl-6,7-dihydro-5*H*-thiazolo[3,2-*a*]pyrimidine (**4d**)

**Figure S8a.
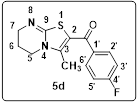
**^1^H NMR spectrum of **4d**

**Figure S8b.** ^13^C NMR spectrum of **4d**

1. **
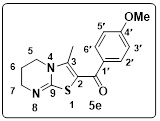
^
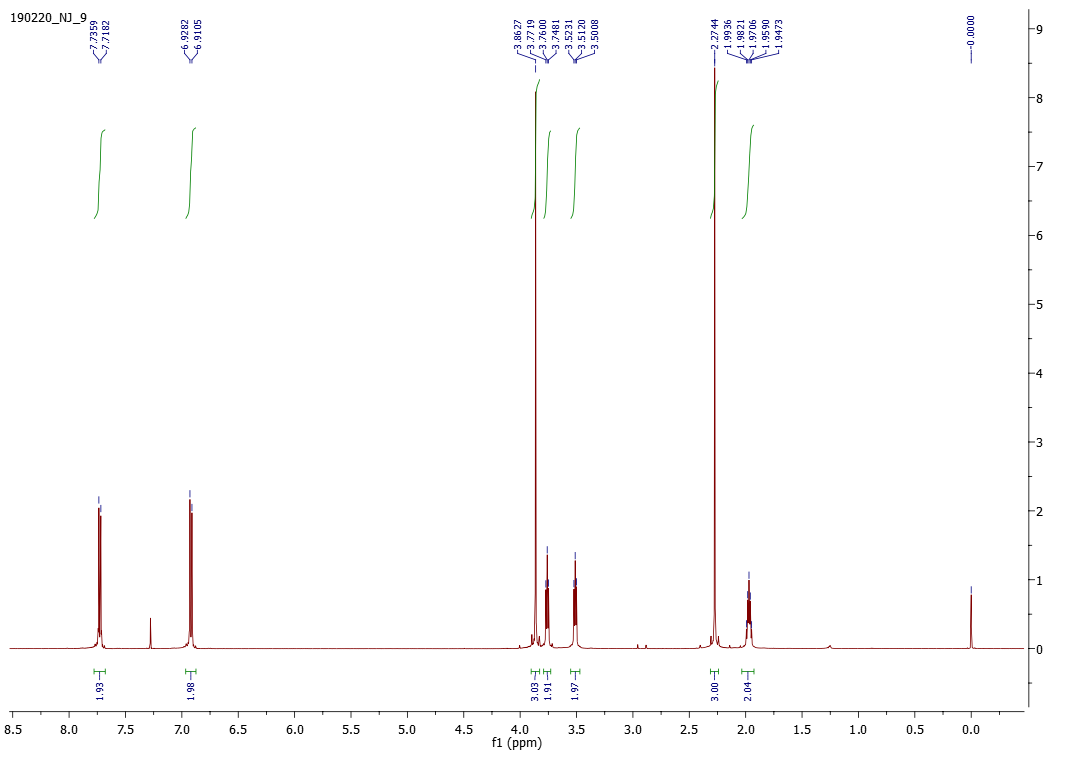
^**2-(4-methoxybenzoyl)-3-methyl-6,7-dihydro-5*H*-thiazolo[3,2-*a*]pyrimidine (**4e**)

**Figure S9a.
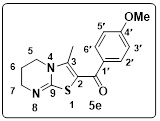
**
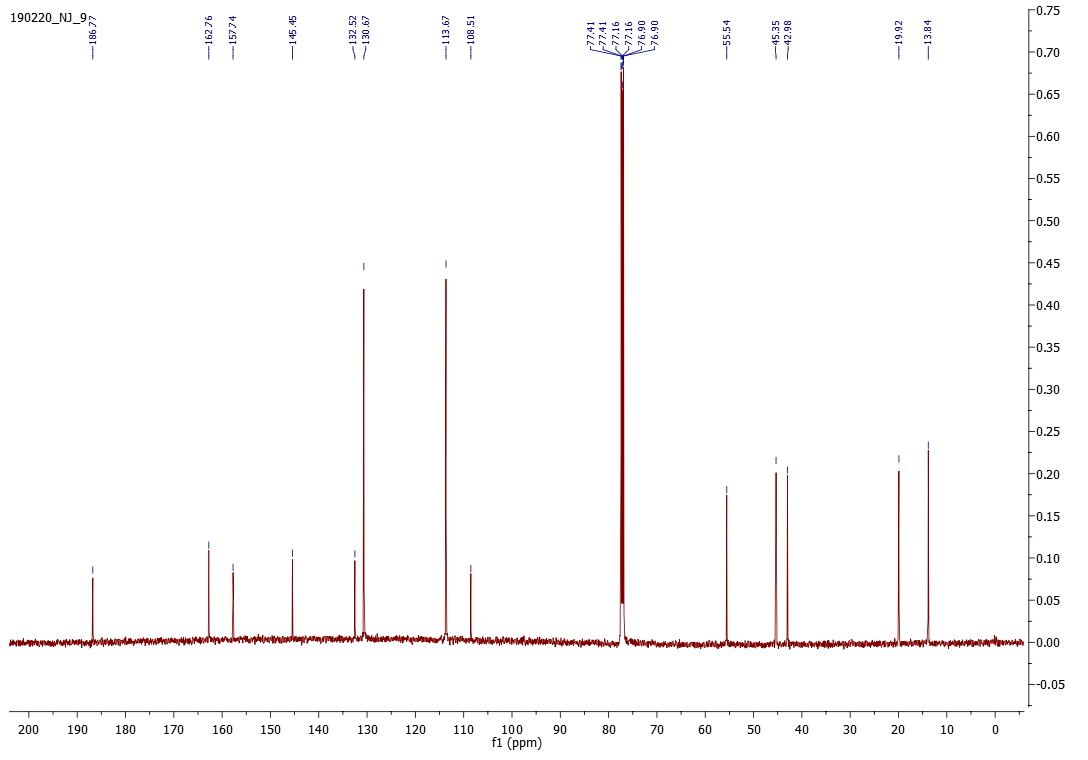
^1^H NMR spectrum of **4e**

**Figure S9b.** ^13^C NMR spectrum of **4e**

1. **
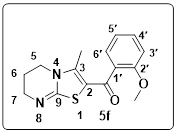
**
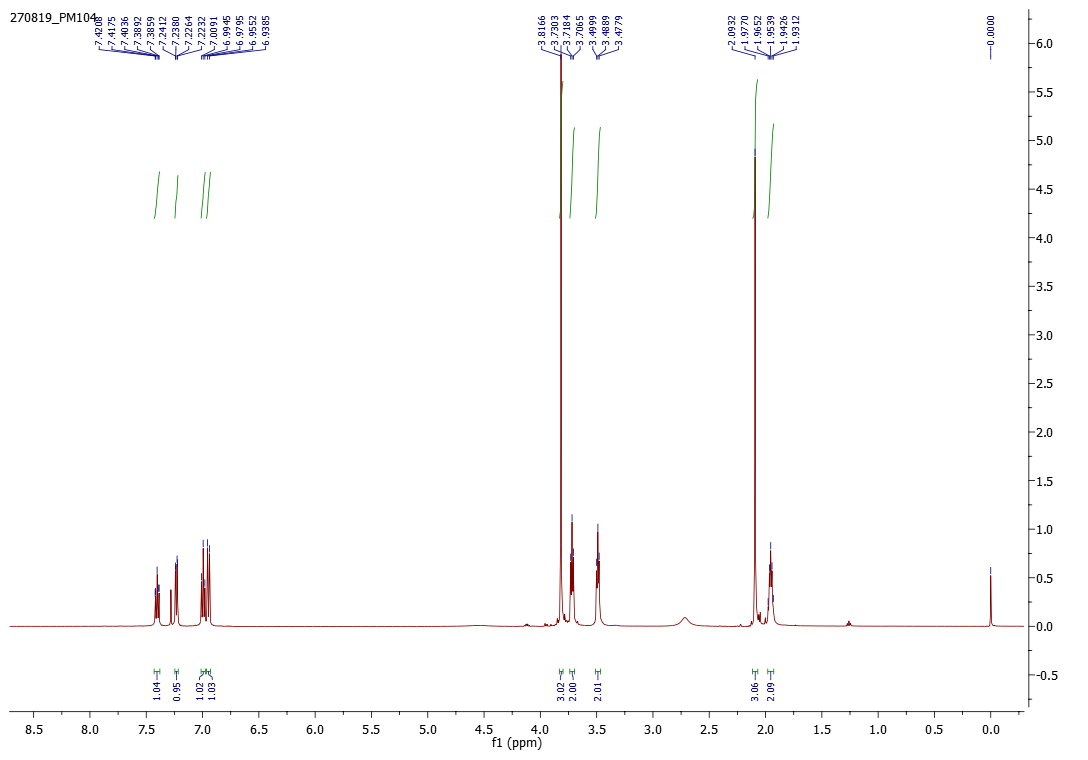
2-(2-methoxybenzoyl)-3-methyl-6,7-dihydro-5*H*-thiazolo[3,2-*a*]pyrimidine (**4f**)

**Figure S10a.
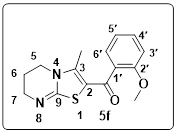
**
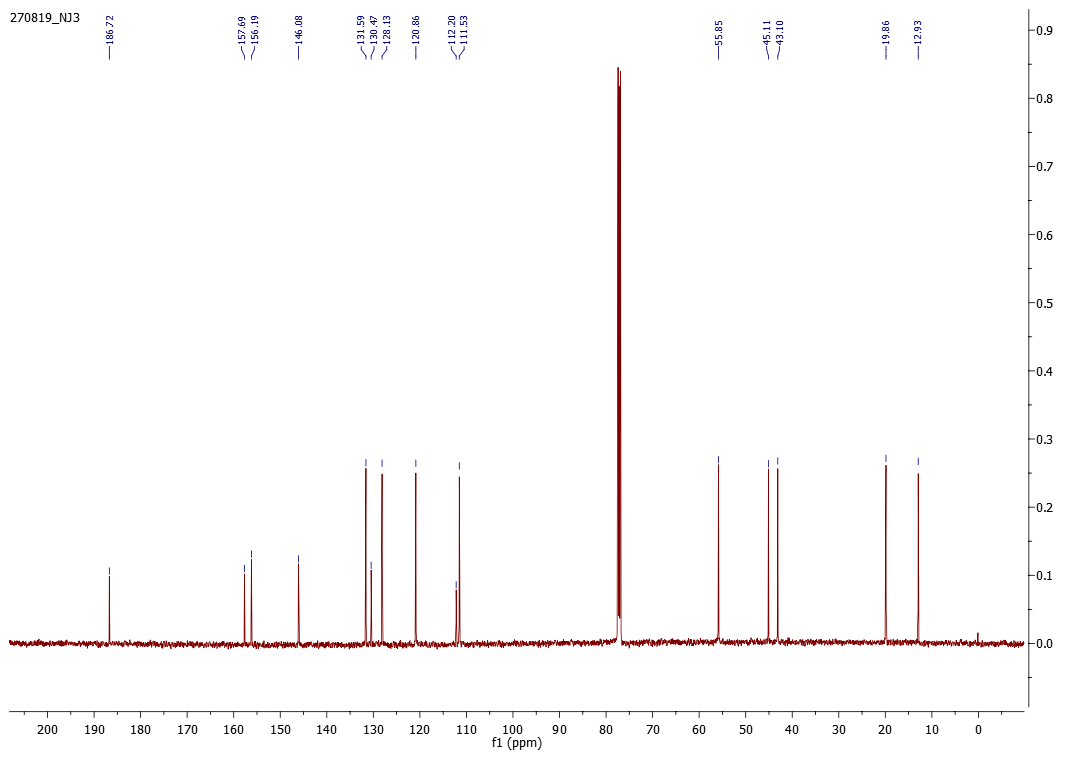
^1^H NMR spectrum of **4f**

**Figure S10b.** ^13^C NMR spectrum of **4f**

1. **
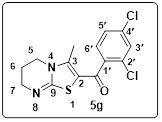
**2-(2,4-dichlorobenzoyl)-3-methyl-6,7-dihydro-5*H*-thiazolo[3,2-*a*]pyrimidine (**4g**)
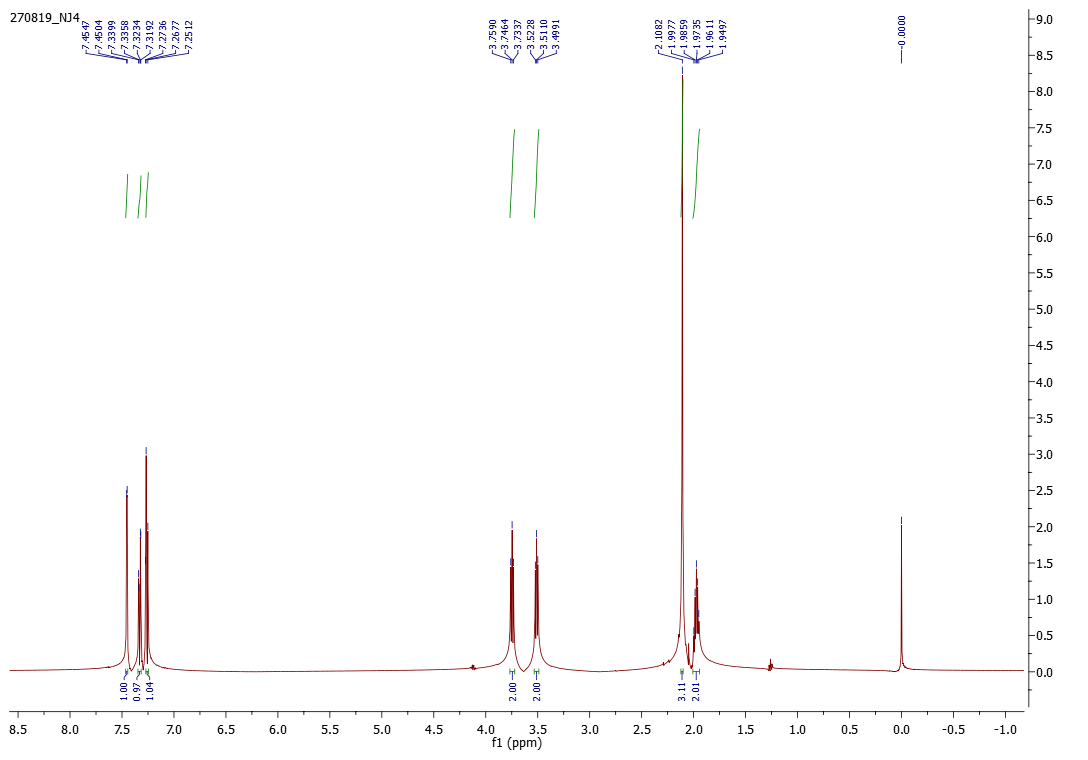


**Figure S11a.
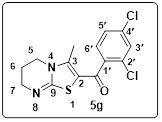
**
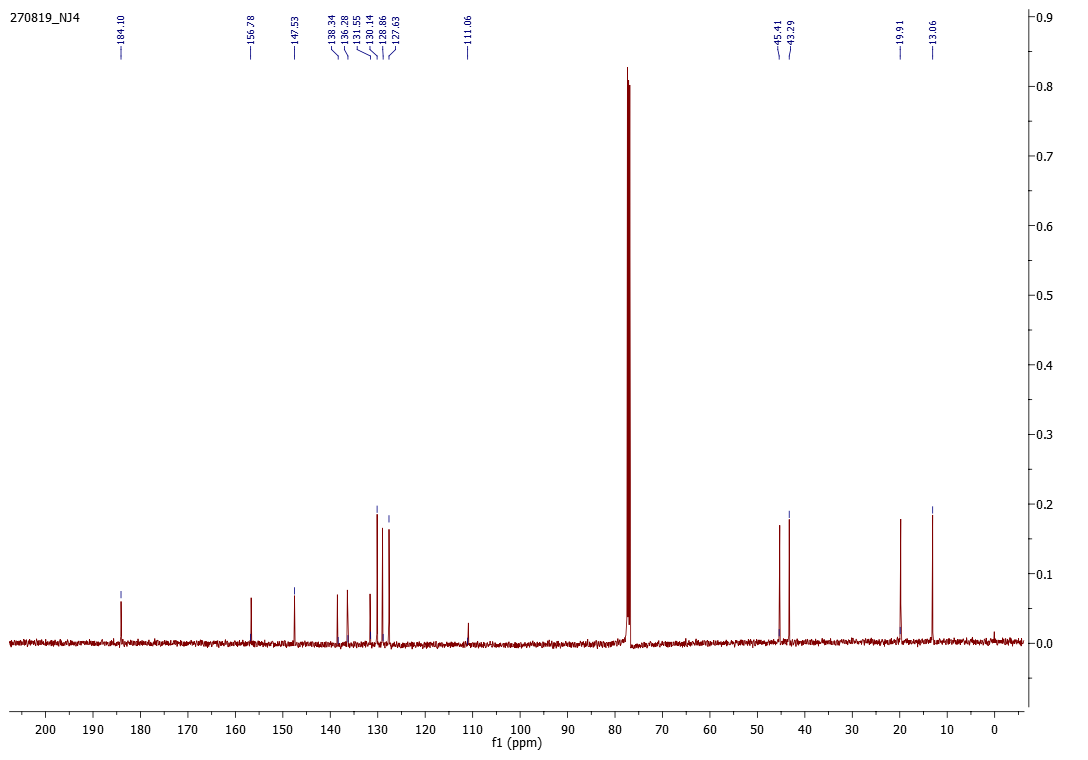
^1^H NMR spectrum of **4g**

**Figure S11b.** ^13^C NMR spectrum of **4g**

1. 2-thienoyl-3-methyl-6,7-dihydro-5*H*-thiazolo[3,2-*a*]pyrimidine (**4h**)


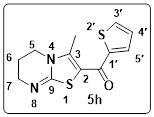

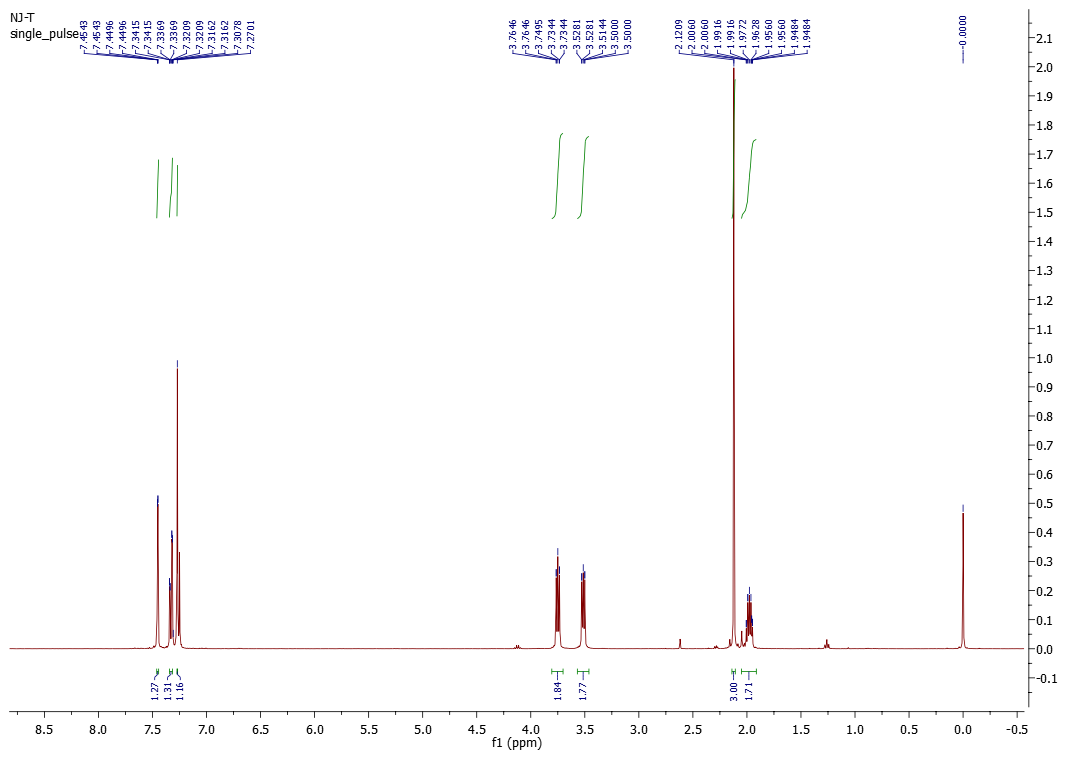


**Figure S12a.** ^1^H NMR spectrum of **4h**


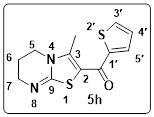

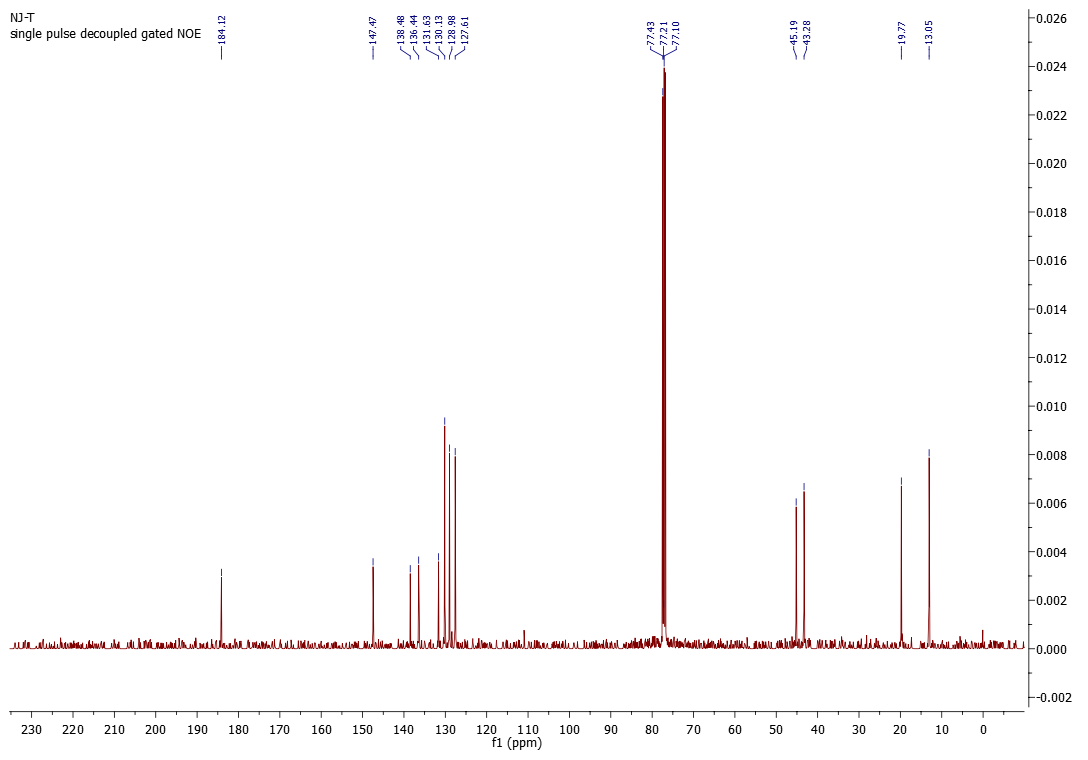


**Figure S12b.** ^13^C NMR spectrum of **4h**

**2D NMR (HMBC & HMQC)**


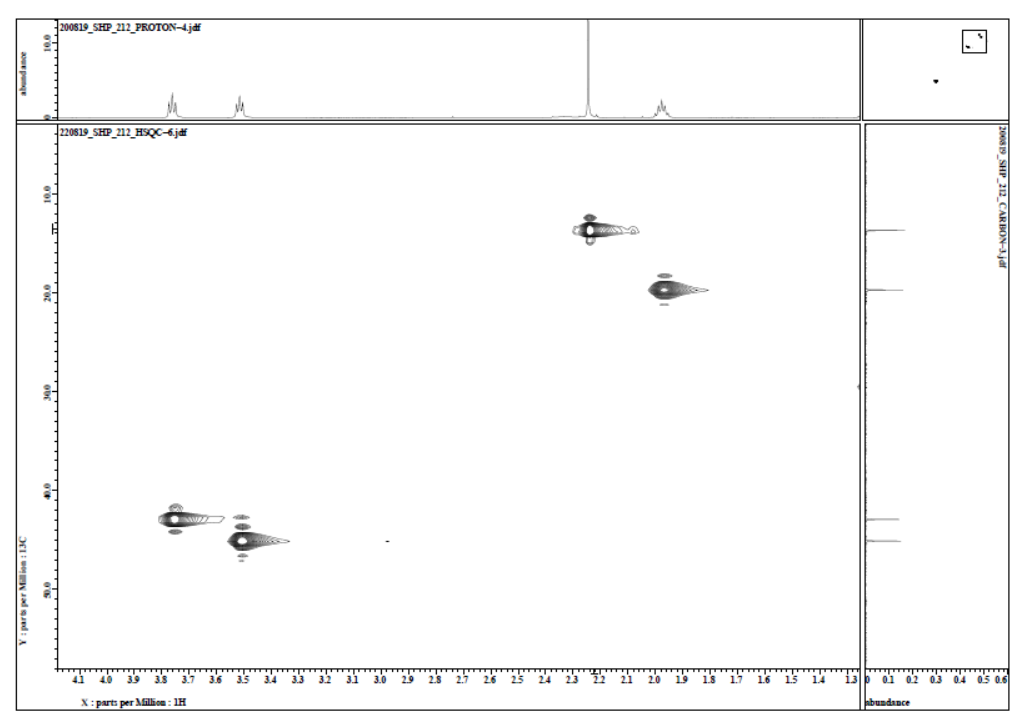


**Figure S13a.** HMQC of **4a**


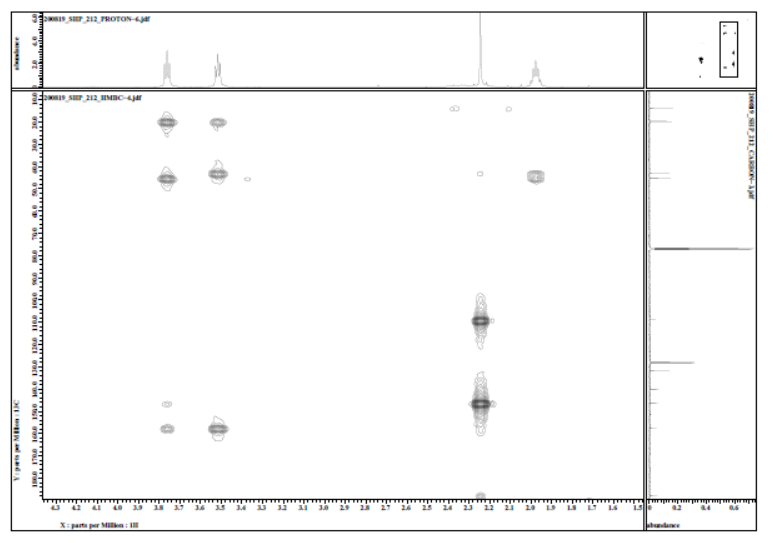


**Figure S13b.** HMQC of **4a**

**Table S1:** 2D NMR correlation results for compound **4a**

| **Chemical shifts**  **(δ in ppm)** | **gs-HMQC** | **gs-HMBC** | **Assignment** |
| --- | --- | --- | --- |
| 187.9 | - | 2.25(3-CH_3_),  7.68-7.66(H-2',6') | CO |
| 157.6 | - | 3.53-3.51 (H7),  3.77-3.75 (H5) | C9 |
| 146.4 | - | 2.25(3-CH_3_) | C3 |
| 140.2 | - | 7.45-7.42 (H-3',5') | C1' |
| 131.9 | 7.53-7.50 (H-4') | 7.68-7.66 (H-2',6') | C4' |
| 128.5 | 7.45-7.42 (H-3',5') | 7.45-7.42 (H-3',5') | C-3',5' |
| 128.1 | 7.68-7.66 (H-2',6') | 7.68-7.66 (H-2',6'),  7.53-7.50 (H-4') | C-2',6' |
| 109.1 | - | 2.25 (3-CH_3_) | C2 |
| 45.3 | 3.53-3.51 (H5) | 2.00-1.95 (H6),  3.77-3.75 (H5) | C5 |
| 43.1 | 3.77-3.75 (H7) | 2.00-1.95 (H6),  3.53-3.51 (H7) | C7 |
| 19.9 | 2.00-1.95 (H6) | 3.53-3.51 (H7),  3.77-3.75 (H5) | C6 |

**Figure S14a.** HMQC of **4f**

**Figure S14b.** HMBC of **4f**

**Table S2:** 2D NMR correlation results for compound **4f**

| **Chemical shifts**  **(δ in ppm)** | **gs-HMQC** | **gs-HMBC** | | **Assignment** |  |
| --- | --- | --- | --- | --- | --- |
| 186.7 | - | 2.09(3-CH_3_),  7.24-7.22 (H-6') | | CO |  |
| 157.6 | - | 3.49-3.47 (H7),  3.73-3.70 (H5) | | C-9 |  |
| 156.1 | - | 3.81 (3'-OCH_3_),  7.24-7.22 (H-6')  7.42-7.38 (H-3') | | C-2' |  |
| 146.0 | - | 2.09 (3-CH_3_) | | C-3 |  |
| 131.5 | 7.42-7.38 (H-3') | 6.95-6.93 (H-5'),  7.00-6.97 (H-4') | | C-3' |  |
| 130.4 | - | 7.24-7.22 (H-6') | | C-1' |  |
| 128.1 | 7.24-7.22 (H-6') | 7.26-7.25 (H-5'),  7.45 (H-3') | | C-6' |  |
| 120.8 | 7.00-6.97 (H-4') | 6.95-6.93 (H-5'),  7.00-6.97 (H-4') | | C-4' |  |
| 112.2 | 6.95-6.93 (H-5') | 7.24-7.22 (H-6'),  7.00-6.97 (H-4') | | C-5' |  |
| 111.5 | - | 2.09(3-CH_3_) | | C-2 |  |
| 55.8 | 3.81 (3'-OCH_3_) |  | | 3'-OCH_3_ |  |
| 45.1 | 3.73-3.70 (H5) | 3.49-3.47 (H7),  1.97-1.93 (H6) | | C-5 |  |
| 43.1 | 3.49-3.47 (H7) | 1.97-1.93 (H6),  3.73-3.70 (H5) | | C-7 |  |
| 19.8 | 1.97-1.93 (H6) | 3.49-3.47 (H7),  3.73-3.70 (H5) | | C-6 |  |
| 12.9 | 2.09 (3-CH_3_) |  | | 3-CH_3_ |  |
|  | | |  | | |

**Figure S15a.** HMQC of **4g**

**Figure S15b.** HMBC of **4g**

**Table S3:** 2D NMR correlation results for compound **4g**

|  | | |  | | |
| --- | --- | --- | --- | --- | --- |
| **Chemical shifts**  **(δ in ppm)** | **gs-HMQC** | **gs-HMBC** | | **Assignment** |  |
| 184.1 | - | 2.10(3-CH_3_),  7.33-7.31 (H-6') | | CO |  |
| 156.1 | - | 3.52-3.49 (H7),  3.75-3.73 (H5) | | C-9 |  |
| 147.5 | - | 2.10 (3-CH_3_) | | C-3 |  |
| 138.3 | - | 7.45 (H-3') | | C-2' |  |
| 136.2 | - | 7.33-7.31 (H-6'),  7.26-7.25 (H-5') | | C-1' |  |
| 131.5 | 7.33-7.31 (H-6') | 7.26-7.25 (H-5') | | C-6' |  |
| 130.1 | - | 7.26-7.25 (H-5'),  7.45 (H-3') | | C-4' |  |
| 128.8 | 7.45 (H-3') | 7.26-7.25 (H-5') | | C-3' |  |
| 127.6 | 7.26-7.25 (H-5') | 7.26-7.25 (H-5'),  7.45 (H-3') | | C-5' |  |
| 111.0 | - | 2.10(3-CH_3_) | | C-2 |  |
| 45.4 | 3.75-3.73 (H5) | 3.52-3.49 (H7),  1.99-1.94 (H6) | | C-5 |  |
| 43.2 | 3.52-3.49 (H7) | 1.99-1.94 (H6),  3.75-3.73 (H5) | | C-7 |  |
| 19.9 | 1.99-1.94 (H6) | 3.52-3.49 (H7),  3.75-3.73 (H5) | | C-6 |  |
| 13.0 | 2.10 (3-CH_3_) |  | | 3-CH_3_ |  |

**HRMS**

**Figure S16.** HRMS of 2-benzoyl-3-methyl-6,7-dihydro-5*H*-thiazolo[3,2-*a*]pyrimidine **(4a)**

**Figure S17.** HRMS of 2-(4-methyl benzoyl)-3-methyl-6,7-dihydro-5*H*-thiazolo[3,2-*a*]pyrimidine **(4b)**

**Figure S18.** HRMS of 2-(4-bromobenzoyl)-3-methyl-6,7-dihydro-5*H*-thiazolo[3,2-*a*]pyrimidine **(4c)**

**Figure S19.** HRMS of 2-(4-fluoro benzoyl)-3-methyl-6,7-dihydro-5*H*-thiazolo[3,2-*a*]pyrimidine **(4d)**

**Figure S20.** HRMS of 2-(4-methoxybenzoyl)-3-methyl-6,7-dihydro-5*H*-thiazolo[3,2-*a*]pyrimidine **(4e)**

**Figure S21.** HRMS of 2-(2-methoxybenzoyl)-3-methyl-6,7-dihydro-5*H*-thiazolo[3,2-*a*]pyrimidine **(4f)**


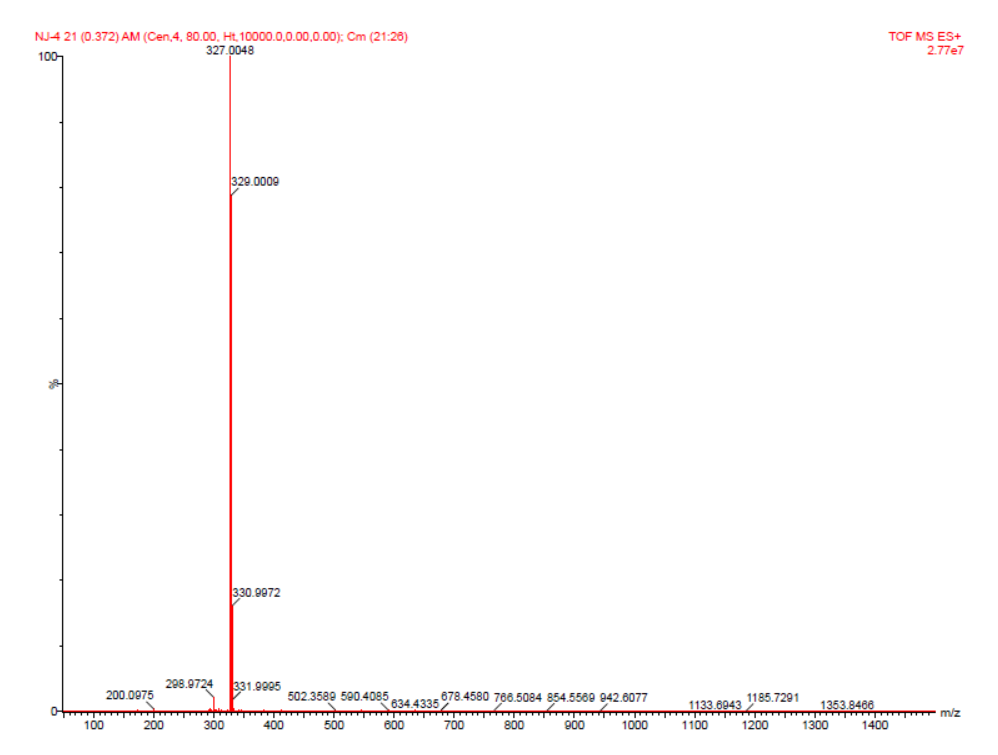


**Figure S22.** HRMS of 2-(2,4-dichlorobenzoyl)-3-methyl-6,7-dihydro-5*H*-thiazolo[3,2-*a*]pyrimidine **(4g)**
